# Biological and Clinical Aspects of Lanthanide Coordination Compounds

**DOI:** 10.1155/S1565363304000111

**Published:** 2004

**Authors:** Sudhindra N. Misra, Minaz A. Gagnani, Indira Devi M., Ram S. Shukla

**Affiliations:** 1 Chemistry Department Bhavnagar University Bhavnagar Gujarat 364 002 India; 2 Central Salt and Marine Chemicals Research Institute Bhavnagar Gujarat 364 002 India; 3 Chemistry Department Nagaland University Nagaland India

## Abstract

The coordinating chemistry of lanthanides, relevant to the biological, biochemical and medical aspects, makes a significant contribution to understanding the basis of application of lanthanides, particularly in biological and medical systems. The importance of the applications of lanthanides, as an excellent diagnostic and prognostic probe in clinical diagnostics, and an anticancer material, is remarkably increasing. Lanthanide complexes based X-ray contrast imaging and lanthanide chelates based contrast enhancing agents for magnetic resonance imaging (MRI) are being excessively used in radiological analysis in our body systems. The most important property of the chelating agents, in lanthanide chelate complex, is its ability to alter the behaviour of lanthanide ion with which it binds in biological systems, and the chelation markedly modifies the biodistribution and excretion profile of the lanthanide ions. The chelating agents, especially aminopoly carboxylic acids, being hydrophilic, increase the proportion of their complex excreted from complexed lanthanide ion form biological systems. Lanthanide polyamino carboxylate-chelate complexes are used as contrast enhancing agents for Magnetic Resonance Imaging. Conjugation of antibodies and other tissue specific molecules to lanthanide chelates has led to a new type of specific MRI contrast agents and their conjugated MRI contrast agents with improved relaxivity, functioning in the body similar to drugs. Many specific features of contrast agent assisted MRI make it particularly effective for musculoskeletal and cerebrospinal imaging. Lanthanide-chelate contrast agents are effectively used in clinical diagnostic investigations involving cerebrospinal diseases and in evaluation of central nervous system. Chelated lanthanide complexes shift reagent aided ^23^Na NMR spectroscopic analysis is used in cellular, tissue and whole organ systems.


### Biological and Clinical Aspects of Lanthanide Coordination Compounds

Sudhindra N. Misra[1], Minaz A. Gagnani[2,*], Indira Devi M.[3] and Ram S. Shukla[2]

[1]*Chemistry Department, Bhavnagar University, Bhavnagar 364 002, Gujarat, India*
[2]*Central Salt and Marine Chemicals Research Institute, Bhavnagar 364 002, Gujarat, India*
[3]*Chemistry Department, Nagaland University, Nagaland, India*

**SUMMARY**

The coordinating chemistry of lanthanides, relevant to the biological, biochemical and medical aspects, makes a significant contribution to understanding the basis of application of lanthanides, particularly in biological and medical systems. The importance of the applications of lanthanides, as an excellent diagnostic and prognostic probe in clinical diagnostics, and an anticancer material, is remarkably increasing. Lanthanide complexes based X-ray contrast imaging and lanthanide chelates based contrast enhancing agents for magnetic resonance imaging (MRI) are being excessively used in radiological analysis in our body systems. The most important property of the chelating agents, in lanthanide chelate complex, is its ability to alter the behaviour of lanthanide ion with which it binds in biological systems, and the chelation markedly modifies the biodistribution and excretion profile of the lanthanide ions. The chelating agents, especially aminopoly carboxylic acids, being hydrophilic, increase the proportion of their complex excreted from complexed lanthanide ion form biological systems. Lanthanide polyamino carboxylate-chelate complexes are used as contrast enhancing agents for Magnetic Resonance Imaging. Conjugation of antibodies and other tissue specific molecules to lanthanide chelates has led to a new type of specific MRI contrast agents and their conjugated MRI contrast agents with improved relaxivity, functioning in the body similar to drugs. Many specific features of contrast agent assisted MRI make it particularly effective for musculoskeletal and cerebrospinal imaging. Lanthanide-chelate contrast agents are effectively used in clinical diagnostic investigations involving cerebrospinal diseases and in evaluation of central nervous system. Chelated lanthanide complexes shift reagent aided $^{23}Na$ NMR spectroscopic analysis is used in cellular, tissue and whole organ systems.

**Keywords:** lanthanides, biological and chemical aspects, coordination compounds, X-ray contrast imaging, $^{23}Na$ NMR spectroscopy.

* To whom correspondence should be addressed.

### 1. INTRODUCTION

Lanthanides, the fourteen chemically similar elements, as such occur only in traces in whole body assay analyses. The amount of lanthanides occurring in different organs shows significant accumulation of these metals, reported in kidney, liver, bones and spleen; however, the remaining organs contain only much smaller concentrations of lanthanides. The amount of lanthanides in eyes has been found to vary in a wide range. The most important and strikingly noticeable part of lanthanide biochemistry is the observation made in a number of studies that the concentration of lanthanides accumulated in different organs varies widely with the progress of different stages in diseases /1/. Webster /2/ reported much higher lanthanide accumulation in infracted cardiac tissues, as compared to the normal ones, as early as in 1965. Esposito and coworkers could locate dramatic upward changes of lanthanide level in the synovial fluid of patients suffering from rheumatoid arthritis of the joints /3,4/. This observation led these workers to propose that lanthanides are "excellent markers" for the "diagnosis and prognosis" of cancer in bones. The lanthanide accumulation has been systematically examined in spleen where it is found that lanthanide level showed regular variation with different degree of infection of the organ in alcoholic persons. Their findings were later on extended to the investigations on liver occurrence of lanthanide, because liver is among the organs which shows great preference for lanthanide accumulation. Their findings suggested that liver which showed great propensity for lanthanide accumulation is damaged and hence becomes less elective in sequestering lanthanides in humans with prolonged alcoholic addiction. This therefore led to significant spilling of lanthanide over spleen, the organ which has a large accumulation of reticuloendothelial cells like liver and hence one could explain higher levels of lanthanides in the spleen of alcoholic persons. The concentration of lanthanides in malignant laryngeal tissues was found to be significantly lower than the normal ones; however, no noticeable lanthanide could be detected in erythrocytic lysate from patients suffering from malignancy of laryngeal tissues /3/. The concentration of lanthanide showed a dramatic spurt (up to 12 fold) in patients with laryngeal carcinoma than that found in normal persons /5,6/.

The toxicity of a non-essential metal ion such as $Ln^{+3}$ can be determined by its degree of deviation from relevant essential metal ion, such as $Ca^{++}$ as reference. The deviation spans the whole range of similarity with respect to $Ca^{++}$. Among the factors determining, how far these metal (Ln) ions deviate from $Ca^{++}$, softness, covalency and redox tendency are the most decisive. The high toxicity of other heavy metal ions or even metals (Hg, Au, Pt, Pb etc) is due to strong deviations in these aspects. Their toxicity is therefore obvious and cannot be avoided. Contrary to this, lanthanides are very similar to $Ca^{++}$ in these aspects. The deviation, however, originates due to deviation in charge, ionic radii and the presence and involvement of inner lying 4f orbitals, and these deviations lead to minor adverse effects, which are related to the level of lanthanides *in vivo* /7,8/.

### 2. COORDINATION CHEMISTRY OF LANTHANIDE, RELEVANT TO THE BIOLOGICAL AND BIOCHEMICAL ASPECTS OF LANTHANIDES

Over the past ten years there has been a resurgence of interest in the coordination chemistry of lanthanide complexes in solution in general, and in aqueous solution in particular. Enthusiasm in this work may be related to the enhanced appreciation of rich functionality of the ground and excited states of lanthanide complexes. The high spin paramagnetism and long electron relaxation times of Gd(III) have made it preeminent among the contrast enhancing agent for "Magnetic Resonance Imaging" (MRI) /7,8/. Related complexes of Dy(III) and Tm(III) with much shorter electronic relaxation times are very effective NMR shift reagents /9/. The controlled modulation of Lewis acidity across the lanthanide series allows the development of the lanthanide complexes exhibiting phosphatase activity, while the redox activity of cerium, samarium, europium and ytterbium may be expected to allow the development of selective oxidants and reductants /10/. Lanthanide complexes in solution exhibit a well defined luminescence which is characterized by narrow emission bands, large stokes shifts and long excited state lifetimes in aqueous solution up to 5 mins which emit in red and green. This has been used in fluoroimmunoassays /11-14/ and shows considerable promise for being used, in luminescence imaging and as sensors for certain bioactive ions /15-17/. Lanthanide(III) also gives characteristic 4f-4f transition bands, which are sharp, narrow and Lapporte forbidden in nature. Under certain conditions created by coordination of certain types of chelating ligands including biomolecular ligands, some of the 4f-4f intra configurational transitions undergo substantial intensification and high sensitivity, towards even minor coordination changes. Such coordinational changes are the outcome of the conformational changes or structural changes which occur during complexation with paramagnetic Ln(III) ions /18-20/. Comparative absorption spectroscopy like luminescence spectroscopy can also be used in certain biological systems to probe the structural, conformational and even changes in the biological activities of biomolecules when these are coordinatively bonded to paramagnetic lanthanides (III) ion /19-22/.

The complexation chemistry of lanthanide ions in solution, especially in aqueous solution, has prompted us and others to progress from the state of getting excited in 1993 /20,21/ to being excited in 2002 /22/. In the past few years several excellent reviews have appeared, detailing aspects of contrast agents and solution dynamics /7,8,23/, biomedical and NMR applications /7,8,24-26/, complex design features and thermodynamic aspects of complex formation /7,27/, development of luminescent lanthanide complexes operating in aqueous media /10-16, 23/, diagnostic and therapeutic use of lanthanide-texaphyrin, and porphyrin complexes /28-31/. A comprehensive analysis of structural aspects of lanthanide-water bond has been published on a number of lanthanide complexes with varying number of coordinated water molecules involving nonacoordinated lanthanides possessing between one and nine coordinated water molecules /31,32/. Much of the current research into the chemistry of lanthanide complexes has been carried out in the solution state and rightly so. It is the properties of these coordination complexes in aqueous solution which are of great importance not only scientifically but also biologically and medically and therefore commercially too.

As a result of different degrees of stabilization, experienced by the 4f, 5d and 6s orbitals, occurring on ionization of the neutral lanthanide metals (La-Lu, = 57-71), lanthanide exist almost exclusively in their trivalent state in coordination complexes and supramolecular assemblies /31,32/. Except for some arene complexes involving bulky substituted benzene, or cyclooctatetrenes, covalency plays only a minor role in Ln-Ligand dative bonds and the nature of coordination sphere is controlled by a subtle interplay between electrostatic interaction and interligand steric constraints /31,32/. Lanthanides, both in crystalline complexes as well as in solutions, show variable coordination number ($6 \leq CN \leq 12$), in which coordination number 9 is the most predominant and several geometries are thus observed, leading to limiting success in the design of molecular architecture with predetermined structure /32/.

For typical non-aquo complexes of lanthanides, all water ligands are not equidistant. Even for aquo ion complex, containing given lanthanide, there is a variation in the length of $Ln\text{-}OH_2$ bond. This is expected for structures in which the coordination environment of water ligands are different, but the structures which have same geometry around metal ion, (and thus the same coordination environment for ligands) have different metal-water bond lengths. Rigid and semi rigid biomolecular receptors may help in, controlling the coordination sphere, according to "Lock and Key" and "induced fit" complex and yielding nonacoordinated lanthanide complexes with tricapped trigonal prism. (TTP) or distorted TTP structures. The number of coordinated water molecules, which can be in $1^{st}$, $2^{nd}$ and $3^{rd}$ spheres, and the relative $Ln\text{-}OH_2$ bond length, in all play dominant role in the binding of lanthanide(III) to the biological substrate, and are decisive factors in finding the biological chemistry and eventually their potential as drug material.

### 3. COORDINATION CHEMISTRY OF LANTHANIDE AND ITS BIOLOGICAL RELEVANCE

A large amount of work reported in literature has given ample proof that the primary site of lanthanide interaction with living cell is only on the external surface, thereby the mechanism of binding of lanthanide to cell membrane requires a closer and sincere attention. No doubt cell membranes are highly complex in structure, the artificial membranes are generally used for understanding the interaction between the phospholipid, the major constituent of cellular membrane with Ln(III) ion /33/. The steps of uptake of Ln(III) involves surface absorption as the first step which can be represented by:

$$\text{Ln(III)} + \text{surface} \rightleftharpoons \text{Ln(III) surface} \quad (1)$$

Alternatively the initial step for the biological system is to provide a chelating ligand L (phospholipid), in case of Ln(III) absorption by cell membrane, in order to capture the metal ion.

$$\text{Ln(III)L} \rightleftharpoons \text{LnL (external surface)} \quad (2)$$

Both steps (1) and (2) must be followed by a passage of the captured Ln(III) deep into the cell:

$$\left.\begin{array}{l}\text{Ln(III) surface L} \rightleftharpoons \text{Ln(III) surface} + \text{L} \\ \text{Ln(III) surface L} \bullet \rightleftharpoons \text{Ln(III)L} \bullet + \text{surface}\end{array}\right\} \quad (3)$$

$$\text{LnL} + \text{L} \bullet \rightarrow \text{Ln L} \bullet + \text{L} \quad (4)$$

The above changes are necessary for the entry of Ln(III) inside the cell. The metal uptake on the cell membrane and strong attachment to the external surface of the bilayer cell membrane can be due to phosphate, which forms strong Ln(III) complex through polar phosphate end as given in Fig. 1. Lecithin (phosphatidyl choline) being the most prominent phospholipid biomembrane, forms 1:2 complex with Ln(III).

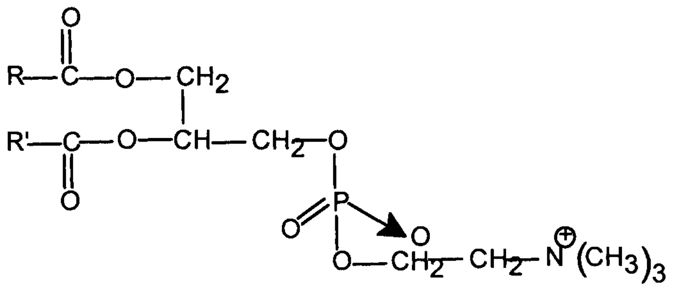

**Fig. 1:** Structure of Lecithin, R and R are long chain alkyl group.

The binding of lanthanide ion occurs at $-PO_2^-$ groups, while the binding of different lanthanide ions for vesicle bilayer is different, most probably due to the presence of interconvertible high affinity sites which are also known as Relaxed (R) sites, low affinity sites and Tense (T) sites. The R/T ratio is fixed and stands around 0.14. The addition of Ln(III) ion increases R/T ratio significantly as a result of conversion of T sites to R sites. This interconversion is mainly due to conformational changes induced by different Ln(III) ion. Paramagnetic Ln(III), due to their NMR characteristics, help in distinguishing outer and inner polar heads of bilayer vesicles. Bentz and coworkers /34/ have reported that in the presence of larger concentrations of Ln(III) ion, their role changed and this initiated the disruption of fusion of unilamellar phosphatidyl serine liposome, most probably by altering the overall charge on the surface of the vesicle. During the process of fusion of vesicles, a leakage of intracellular contents took place. No doubt the role of Ln(III) is much faster and stronger than the role played by Ca(II) in the fusion of vesicle where the leakage of intracellular contents took place /34/. El-Fakahany *et al.* used EM (electron microscopy) EDAX (energy disperse analysis of X-rays) studies in explaining Ln(III) binding to biological membranes and their findings conclusively remarked that the distribution of Ln(III) was basically irregular and was in the form of "clusters" named as "Hot spots". These hot spots or confined areas of lanthanide accumulation most probably play the role of Ca(II) channel around receptor /35/.

The above findings have been found very effective in exploring lanthanide compounds (salts, complexes as well as coordinated chelates) in drug development as well as during diagnosis and prognosis of diseases, like multiple-sclerosis, atherosclerosis, cerebrospinal and cardiovascular, and oncological diseases /33, 34, 36, 37/. Very recently immensely useful investigations have been carried out, involving lanthanide induced perforation of cell membrane. In gene recombination, the critical step that is necessary is to promote transformation of plasmid in bacteria by incubation with $CaCl_2$. The elevation in the permeability induced by Ca(II) was the main factor in perforation mechanism /38, 39/.Wang *et al.* /40,41/, reported that the perforation of membranes was caused by Ln(III) even when these are administered in low concentration.

Huang *et al.* /42/ found Ln(III) ions like Ca(II), when applied Ln(III) enhanced the transformation of plasmid pBR 322 and PUC 18 in E.coli and highest transformation required a dose as low as $10^{-5}$ M of Ln(III). No doubt higher concentration of lanthanide application inhibited plasmid transformation. Canada *et al.* /43,a/ reported earlier that Ln(III) binding to cell surface is always accompanied by significant physiological changes like rapid increase in membrane potential especially in Ehrlich ascite tumor cells and this type of lanthanide ($Tb^{3+}$) binding also led to substantial changes in electrophoretic behaviour. However, in their latter publication Canada *et al.* /43,b/ found that Tb(III) increases significantly the intracellular accumulation of cisplatin. Their observation was soon supported, though indirectly, by permeability increase induced by Ln(III) reported by Wang *et al.*, who proposed the mechanism for perforation induced by metal ion like Ln(III). Hence this has been used in the leaking of hemoglobin from erythrocytes by the presence of lanthanide(III) salts or compounds /42/. Their findings /42-44/ showed that lanthanide aquo complexes were quite effective in perforation of erythrocytes. Complexation interaction is a triphasic process, with perforation occurring in the second stage. This stage was characterized by sustainable and recoverable hemolysis. EDTA washing could lead to resealing of the membrane of erythrocytes. These findings could prove that Ln(III) complexation with cell surface-active centre involves a predominantly electrostatic interaction /43-45/. There are two types of pores, domain and crater shaped pores, which result due to different concentrations of Ln(III) used during investigations. Ln(III) binding incurs conformational changes followed by aggregation of membrane proteins /46-48/. Since lanthanide(III) ions in aqueous medium are always in the form of a nonacoordinated stereochemistry and in biofluids these undergo multimetal multiligand complexation due to the presence of endogenous metal ions and physiological ligands. Therefore even when Ln(III) ions are used, to alter the biological properties in Ln(III) and biological substrate interaction, complexation is undergone with the binding sites of the biological substrate by partial substitution of coordinated water molecules surrounding tervalent lanthanide(III) in $[Ln(H_2O)_9]^{3+}$. The extent of substitution depends on several factors like pH, composition and *in vivo* chemical environment. The binding sites of the biological substrate which are preferred by Ln(III) for complexation involve donor sites $O \geq F > Cl > N < S$. The inherent strong oxyphilicity of lanthanide causes the interaction binding sites being COOH, OH (phenolic) OH (hydroxylic), O (carbonyl), N (amino, imido, imino), S (sulphydryl). Nitrogen sulphur donor sites of biomolecules also enter into complexation when Ln(III) undergoes chelation.

A number of techniques like Atomic Force Microscopy (AFM), $^{1}H$, $^{13}C$, $^{31}P$ NMR, FT-IR luminescence and absorption spectroscopy have been found very useful in such studies. Lanthanides consuming, Reactive Oxygen Species (ROS), which are mainly the oxygen derived free radicals and peroxides, are the mediators of a number of degenerative diseases. The 'antioxidants' are excellent substances used as drugs for the treatment of degenerative diseases. Tocopherol, ascorbates and a number of other organic compounds are considered as components of such drugs due to their antioxidant property shown towards ROS induced degenerative diseases. Lanthanides are considered of high potential because of their inherent antioxidant properties /22/. Wu *et al.* /49/ found $LaCl_3$ effective in inhibiting silica induced lipid peroxidation of lung macrophagus and smaller doses of some other lanthanide chlorides inhibited lipid peroxidation in rat lung. Wang *et al.* found almost all lanthanide compounds, especially chlorides, quite effective in inhibiting $H_2O_2$ mediated peroxidation of liposomes; however, when tertbutyl hydroperoxide was used to mediate the peroxidation, lower lanthanides inhibited while the higher lanthanides promoted peroxidation. Interestingly

Ln(III) lost reactivity of peroxides when they were bound to membrane. The prior lanthanide(III) became more sensitive to oxidation attack. The lanthanide inhibiting ROS involves strong oxyphilicity inherent in lanthanides, because of the availability of oxygen sites on these free radicals, makes them excellent targets for Ln(III) coordination (attack). This causes lanthanide to play the role of scavenger of reactive oxygen species, therefore presenting good potential for lanthanide as a future drug for a number of degenerative diseases due to ROS. No doubt the involvement of lanthanide in ROS removal is quite different from the inhibition of ROS by organic compounds like Tocopherol, Ascorbate etc. Most of the organic antioxidants scavenge free radicals by single electron exchange with radicals and thus transform themselves into radicals, hence acting as "pro-oxidants". $Ln^{3+}$ very easily interacts with either free radicals or peroxides but is not transformed as radicals. However the mechanistic understanding about the role of Ln(III) as scavenger of antioxidant is very meager.

The lability of lanthanide complexes, strong oxyphilicity, very fast water exchange reaction, non-directionality of lanthanide ligand bond and varying coordination number, all contribute towards lanthanide interaction with biomolecules. The ionic size of Ln(III) varies from one lanthanide to another lanthanide; in addition, the ionic size of a particular lanthanide also varies significantly with the coordination number. Some salient properties of calcium and lanthanide are listed in Table 1.

Smaller size of chelating biomolecular ligand can even suit larger lanthanides with lowered coordination number. Similarly small lanthanides can expand their coordination number and can form stable chelates with larger biomolecules. This can explain the different coordinating power (also biological behaviour) of different lanthanides under different physiological conditions.

**Table 1**

Salient properties of calcium and lanthanide

| Property | Ca(II) | Ln(II) |
|---|---|---|
| Coordination number | 6-12 reported<br>6 or 7 favoured | 6-12 reported<br>8 or 9 favoured |
| Coordination geometry | Highly flexible | Highly flexible |
| Donor atom preference | O ≫ N ≫ S | O ≫ N ≫ S |
| Ionic radius (A)[a] | 1.00 – 1.18 (CN6 – 9) | 0.86 – 1.22 (CN6 – 9) |
| Type of bonding | Ionic | Ionic |
| Hydration number b | 6 | 8 or 9 |
| Water exchange rate constant ($s^{-1}$) | $5 \times 10^8$ | $5 \times 10^7$ |
| Diffusion coefficient ($cm^2/s \times 10^5$) | 1.34 | Ln(III), 1.30 |
| Crystal field stabilization | None | Negligible |

### 4. LANTHANIDE ALTERING THE STABILITY OF MICROTUBULES

Significant disorganization in cytoskeleton including microtubules and microfilaments is a phenomenon of common occurrence in tumor cells and apopto sized cells. Microtubules undergo stabilization and repair and this occurs during the action of some anticancer drugs like TAXOL. Depolymerization of cytoskeleton is one of the most relevant steps in the apoptosis process. Lanthanide compounds have been shown to influence the stability of microtubules. Xiao *et al.* /50/ found mixed lanthanide compounds, increasing the amount of orderliness of microtubules in PAMC82 cells. Soto *et al.* /51/ found different lanthanides showing different behaviour and this was ascribed to similarity of Ln(III) predominantly with $Ca^{++}$ or similarity of Ln(III) with $Mg^{2+}$, because $Ca^{++}$ ion was found to destabilize microtubules contrary to the strengthening of microtubule activity induced by $Mg^{2+}$. The lower lanthanides behaved more like $Ca^{++}$, because of their larger size and hence propensity of exhibiting relatively higher coordination number. Heavier lanthanides like Tb, Dy, Ho being smaller in size behaved more like $Mg^{2+}$ having more potential for strengthening the microtubules. Soto *et al.* attributed the role of Ln(III) and different behaviour of different lanthanides to their effect of GTPase activity of tubulin. The microtubule formation tubulin-GTP-$Mg^{++}$ system is a multiple step process, where the role of $Mg^{2+}$ is very important because it binds to tubulin and thus modulating the conformation to favour self association of tubulin as shown in Fig. 2.

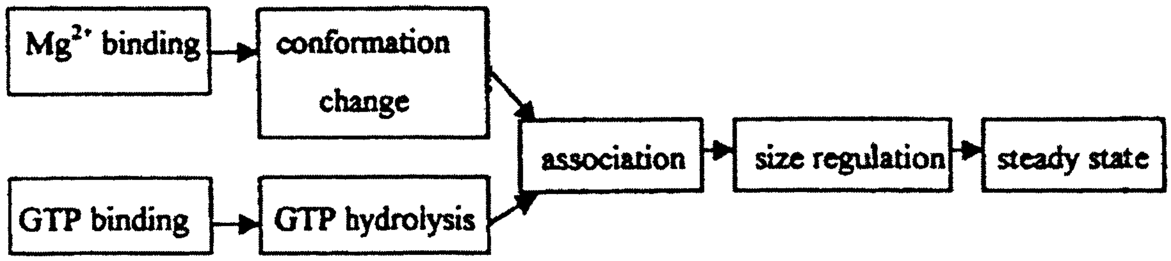

**Fig. 2:** Probable steps involved in the formation of microtubule.

As we have explored the complexation of mononucleotides with paramagnetic lanthanide using nucleotide, mono-, di- and triphosphates, in aqueous and in aquated organic mediums at pH as low as 1.00 to as high as 6.5, using 4f-4f transition spectroscopy $^{1}H$ NMR and $^{31}P$ NMR spectroscopy. We have found that different lanthanides showed different stability of Ln-mononucleotide complexes. The nature of the nucleoside moiety and the number of phosphate groups significantly affected the degree of complexation. The size of Ln(III) ion also played an important role.

Comparative absorption spectroscopy, involving electric dipole Lapporte forbidden 4f-4f transitions, have shown that different mononucleotides showed different affinities towards Ln(III). In general the binding of Ln(III) with nucleotides derived from pyrimidine bases is weaker than the binding of Ln(III) with nucleotides derived from purine bases, irrespective of the nature of the experimental conditions. We have also observed that in aqueous medium lanthanide interaction with mononucleotides shows the presence of both syn and anti conformation. The increase in the organic solvent percentage resulted in the shift in the equilibrium towards anticonformations of the Ln-nucleotide complex /52-55/.

Our studies also showed that in aqueous solutions, Ln(III) initiates the hydrolysis of NTP (nucleotide triphosphate) and this reaction was appeared to be dependent on $H^+$ ion concentration and the nature of lanthanides /55-58/.

The important steps in the association of tubulin are GTP hydrolysis and tubulin binding to GTP, and both of these activities are governed by $Mg^{2+}$. With association process, setting in the size of regulatory effect proceeds and also consequently controls the shape and size of microtubule. $Ln^{3+}$ when administered in small doses behaves like $Mg^{2+}$ supporting the association of tubulin. However when administered in high doses, $Ln^{3+}$ interferes with the assembly, by distorting the protein conformation, altering crosslinking and consequently destabilizing the polymers.

### 5. LANTHANIDES AS EXCELLENT DIAGNOSTIC AND PROGNOSTIC PROBES IN CLINICAL DIAGNOSTICS AS WELL AS ANTICANCER MATERIALS

In 1931 Maxwell and coworkers /59/ used an aqueous solution of lanthanum chloride for treating cancer by administering $LaCl_3$ solution intraperitoneally. However, it was only the work of Anghileri and coworkers which could successfully demonstrate the strong inhibitory effects of $LaCl_3$ and other lanthanide compounds on the growth of sarcoma tumors in rats. Excellent work has come out of Anghileri's laboratory, on lanthanide compounds and complexes in cancer research as a diagnostic and prognostic probe /60-63/. These workers used Ln(III) as an adjunct to the distraction of tumors by using a combination of the complexes of two different lanthanides specially derived form hydroxy carboxylic acids for treating animals and also in some cases involving humans suffering from Yoshida Sarcoma. The results were found astounding /63/.

In our preliminary studies we have used a combination of two or even three different lanthanide complexes derived from citric and mandelic acids on rats with Yoshida Sarcoma and found encouraging results /64, 65/. The synthesis and reactivity of these citrates, mandelates and tartarates have been reported from our laboratories in 1966. Though our study is quite preliminary, the results are very promising. Lanthanide citrates and mandelates, when administered along with drug hematoporphyrins, showed drastic reduction of growth of Ehrlich ascite cells, much better than that obtained by using hematoporphyrin alone. We attributed this to much improved absorption of the drug due to the presence of lanthanide coordination complexes /66/.

The complexes of lanthanides are getting more and more applications in cancer therapy and the most important of these are those derived from poly (aminocarboxylic) acids. The formation constants of the lanthanide chelates with these acids are of the order of $10^{20}$ to $10^{25}$, which enables them to remain intact, while diffusing into extracellular spaces with rapid clearance through kidneys. Due to the high thermodynamic stability and extreme kinetic inertness of these poly (amino carboxylates) of Ln(III), the intact excretion enhances, thereby lowering considerably the body retention of chelated Ln(III) complexes.

These days diagnostic imaging procedures /67, 68/ are a routine part of modern medicine and are useful in performing the initial diagnosis, the planning of the treatment and post treatment evaluation.

### 6. Ln(III) COMPLEXES BASED X-RAY CONTRAST IMAGING

Even with the recent phenomenal growth of magnetic resonance imaging (MRI) and ultrasound procedures, X-ray imaging studies remain even today the workhorse of modern radiology. Currently 75-80% of all diagnostic imaging procedures, as listed in Table 2, are X-ray related /68-73/.

**Table 2**

X-ray diagnostic procedures by body system

| Body System | Diagnostic Procedures | Technique | Share % |
|---|---|---|---|
| Vasculature | Angiography, arteriography (arteries), venography (veins), ventriculography and interventional angiography | a) intravenous or intra-arterial administration of soluble contrast media during imaging<br>b) patient is catheterized by administering CA near the region of interest<br>c) interventional procedures include vessel remodeling procedure such as angioplasty, atherectomy during angiographic visualization<br>d) angiographic procedure | 17% |
| Organs | Brain CT, abdominal CT, liver CT, hepatosplenography, pyelography, cholecystography | General i.v. administration of contrast agent prior to image acquisition, often before and after the CA administration images are taken. | 54% |
| Spinal canal | Myelography (spine) and cisternography (brain) | Direct injection of CA into spinal canal or subarachnoid space | 2% |
| Urinary tract and bladder | Retrograde pyelography and urethrography | Contrast administration through catheter placed in bladder | 3% |
| Joints | Arthrography and diskography | CA is administered directly into joints | 2% |
| Uterine cavity and fallopian tubes | Hysterosalpingography | Contrast agents administered to uterus and/or to fallopian tubes | 2% |
| Rest | | | 20% |

To better delineate soft tissues regions, such as cardiovascular system and cerebrospinal systems, safe and efficient X-ray contrast agents (also called radiographic contrast agents, radiopaque agents or roentgenographic agents) were long sought after. Contrast agents are a class of pharmaceuticals that, when administered to a patient, enter and pass through anatomic regions of interest to provide transient contrast enhancement. These contrast enhancing agents are then completely excreted renally from patients without being metabolized.

A metal chelate complex is a coordination compound of metal ion with a chelating ligand. The most important property of the chelating agent is its ability to alter the behaviour of metal ion with which it binds in biological systems. The biodistribution and excretion profile of these metal ions will be markedly modified on chelation, leading generally to lowering the *in vivo* absorption of metal ion. The chelating agents, especially amino poly carboxylic acids, (EDTA, CDTA, HEDTA, DTPA, DCTA etc) and DOTA, HP-DO3A etc. being hydrophilic, increase the proportion of their complex excreted from complexed metal ion from biological systems. Conversely a lipophilic chelating agent such as EOB-DTPA (ethoxy benzyl DTPA), Fig. 3, results in its metal complexes being taken up by the liver and bile and excreted fecally.

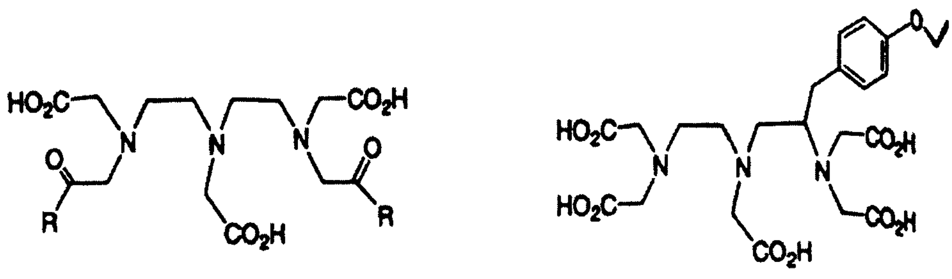

$H_5$EOB-DTPA

R = OH, $H_5$DTPA; R = NHMe, $H_3$DTPA-BMA

**Fig. 3:** Chelating agents DTPA and EOB-DTPA.

The first testing of $(NMG)_2$GdDTPA (NMG-N-methyl glucamine) as an X-ray contrast agent in humans occurred serendipitously, almost paralleling the first human experience with iodinated contrast /74/. The easy availability of $(NMG)_2$GdDTPA for human studies /75-78/ has made additional diagnostic /79-84/ investigations possible. $(NMG)_2$GdDTPA has found wide use in digital substraction angiography (DSA), for diagnosing the patients suffering from renal inefficiency or allergy with iodinated contrast agents. The CA (contrast agents) is yielding adequate diagnostic information. Renal functions remained stable. GdDTPA-BMA (GADODIAMIDE-OMNISCAN) is an approved contrast agent prepared from a linear chelating group DTPA-Bis (methyl) amide ligand. The ligand contains only three anionic groups and hence the CA is neutral or nonionic in nature. The pharmaco-kinetic and biodistribution profile of Gd DTPA-BMA is similar to $(NMG)_2$GdDTPA. Gadodiamide, an extracellular agent, is rapidly excreted renally, with elimination half time around 70 min. A computed tomography (CT) phantom study showed that GdDTPA-BMA produced the same level of X-ray contrast enhancement as that of produced by $(NMG)_2$GdDTPA. The utility of GdDTPA-BMA as X-ray contrast enhancing agent was very effective in arterial angiography specially in the diagnosis and treatment evaluation of renal artereostenosis in patients suffering from renal insufficiency /85-87/. The images produced by GdDTPABMA depicted renal artery occlusions and provided far better delineation of the renal vessel.

GdEOB-DTPA is currently in the late stages of clinical testing as an MRI agent for liver and spleen. The addition of lipophilic ethoxy-benzyl group to the carbon backbone of DTPA profoundly altered the pharmaco-kinetic and biodistribution properties of $(NMG)_2GdDTPA$. Following its administration, Gd EOB-DTPA rapidly distributes into the extracellular fluid space. However, unlike $(NMG)_2GdDTPA$, which is exclusively excreted through the kidneys, Gd EOB-DTPA is taken by hepatocytes (pently) during its short plasma residence time, through the organic anion plasma membrane transport system to provide a clinically very useful MRI and X-ray contrast enhancement of the liver and spleen before being fecally excreted /88,89/.

GdHP-DO3A (GADOTERIDOL-or ProHance) and GdDO3A-butrol (GADOVIST-gadobutrol) are both neutral gadolinum chelates used effectively as contrast agents in MRI as well as in CAs for CT. The low toxicity of these CAs permits one to use even the higher doses of these CAs for much enhanced imaging safely, which helps in imaging the abnormalities, even in their nascent stages of cancer and thus this early detection makes planning of treatment very easy. Both of these helped in detecting brain tumors even at very early stages using CT. The pharmaco-kinetic profile of Gd HP-DO3A and Gd DO3A – butrol are similar to that of $(NMG)_2$ Gd DTPA. Once injected, they rapidly distribute from vascular compartment in the extra cellular fluid space and predominantly excreted through the renal system. Their high solubility allow even preparation of a solution up to one molar concentration. GdDO3A butrol can also be developed as an alternative X-ray contrast agent.

### 7. LANTHANIDE CHELATES WITH POLY(AMINO CARBOXYLIC) ACIDS AS "CONTRAST ENHANCING AGENTS" FOR MAGNETIC RESONANCE IMAGING (MRI)

Like platinum in cancer therapeutics and technetium in cardiac scanning, the unique magnetic properties of gadolinium(III) ion placed it right in the middle of revolutionary development in clinical medicine Magnetic Resonance Imaging (MRI). Up to 1987 only 39 papers were published on Gd(III)DTPA in MRI. In the late 90's (1999) approximately 600 references appear each year on MRI using Gd chelates as contrast enhancing agents /90/. MRI, having different properties of CA, is relied upon by neurologist, cardiologists, urologists, ophthalmologist and even gastroenterologists world wide, in search of new ways to visualize functional changes in the body.

Gd(III) ion is too toxic at the level used in MRI and it cannot be injected as such. DTPA is the choice of ligand because it forms very stable chelate with $Gd^{3+}$ and facilitates renal excretion. Cyclic polyaminocarboxylates of Gd(III) like DOTA are less favoured because the preparation of 1, 4, 7, 10-tetra-aza cyclodecane was difficult.

Macrocycles derived from DOTA and substituted in tetraaza cycle or on the acetate arms have been prepared either by linking chelates to a macromolecule or by achieving a higher hydrophobicity. The increased hydrophobicity favours hepatobiliary uptake and excretion and this leads to better imaging of kidney and liver /91/. There is only one water molecule in the first coordination sphere of the complexed Gd(III) with tetra-acetic ligands derived from cyclen and syntheses have been done to obtain chelates with a higher degree of hydration even if it leads to a decrease in the thermodynamic stability of the complexes. For that purpose, cyclen can be selectively substituted on one, two or three nitrogen atoms in nearly all possible stereochemical arrangements. The preparation of 1,7-disubstituted cyclen is quite easy. Ligands like DO2A

are readily obtained /92/ and the trade-off between a higher hydration number, because of a more open structure and a lower stability, because of a smaller number of coordinating groups, which might not be too unfavourable since DO2A has been reported to form stable lanthanide chelates /93-96/. Weisman *et al.* /94/ obtained a more rigid chelate of DO2A type by bridging tetra-aza ring by ethylene or propylene group. DO(EN)2A (Fig. 4) is one among many examples illustrating the remarkable flexibility that has been achieved for modification of structure of cyclen.

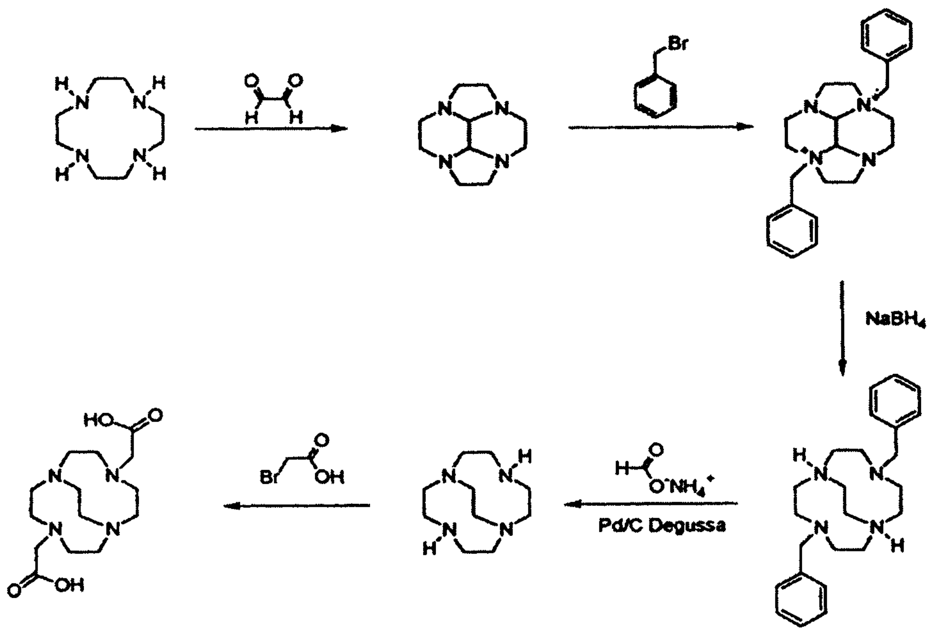

**Fig. 4:** Synthesis of DO(EN)2A.

The kinetic inertness and thermodynamic stability of new and modified DOTA ligands are of the utmost importance, since stability affects directly the toxicity of the Gd(III) chelates. DOTA itself forms exceedingly stable lanthanide chelates, presumably because of the tetra-aza cycle which is able to adopt its most stable conformation, a square conformation, in which all lone electronic pairs of nitrogen are directed toward metal ion. The kinetics, of formation and even more remarkably, of dissociation are extremely slow, which is a unique feature in the coordination chemistry of 4f elements.

Since during the progress of disease, the water contents in the tissue show remarkable variation, NMR signals are related to the water content in the tissues when MRI is being investigated. The increased number of coordinated water molecules found in the contrast enhancing agent makes better contribution towards much improved delineation of tissues during imaging. A large number of Gd(III) chelates have been synthesized and used as contrast agents in clinical trials. Fig. 5 shows some well known commercially available contrast agents showing the coordination environment around Gd(III).

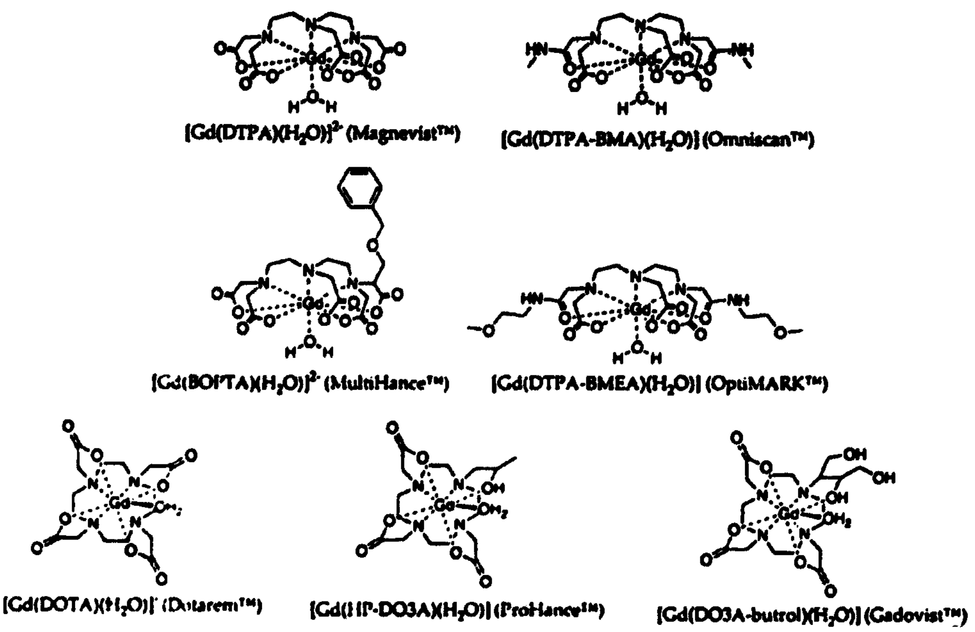

**Fig. 5:** Coordination environment around Gd(III).

Gd DTPA, the most widely used contrast agent, has one coordinated water molecule present in the 1st coordination shell and it is this water molecule which makes this chelate an effective CA for MRI investigations. A number of chelating ligands have been synthesized. All structures provide a nona coordinated environment for Gd(III) bound with three nitrogen and five monodentate carboxylate oxygen of DTPA ligands. The resultant Gd(III) complexes have a structure of either distorted TTP (tricapped trigonal prism) or CSAP (mono capped square antiprism) /97,98/.

Several structures (Fig. 6) of DTPA–bisamide complexes, [LnDTPA-BMA ($H_2O$)], [Ln(DTPA-BEA) ($H_2O$)] and [Ln(DTPA-BBA) ($H_2O$)] have been determined. All these complexes involved nonacoordinated amide oxygen and one coordinated water molecule. There are four possible mode of configurations for the placement of amide groups (syn, cis, anti and trans), which are depicted in Fig. 7.

A number of isostructural Ln(III) complexes of 15-DTPA-EAM are crystallized as binuclear centrosymmetric structures with two Ln(III) ions located between two ligand molecules. The nona-coordinated Ln(III) were coordinated to an amide oxygen, two carboxylate oxygens and amide nitrogen donors from one ligand, and an amide oxygen, a carboxylate oxygen and an amine nitrogen from second ligand. The coordination sphere around Ln(III) is completed by a water molecule /99,100/. The ring is expanded with 16-DTPA-PAM /100/, 16 DTPA-HPAM /101/ or 17 DTPA-BAM /102/ as ligands. Several structures of DTPA-BMA, DTPA-BBA, DTPA-BMEA are given in Fig. 8.

The ligands 16 DTPA-PAM, 16 DTPA-HPAM or 17 DTPA-BAM (Fig. 9) can wrap around the metal center, which lead to the formation of mononuclear structure with tervalent Ln(III). The geometry around metal centre is TTP with amide sitting in a syn configuration.

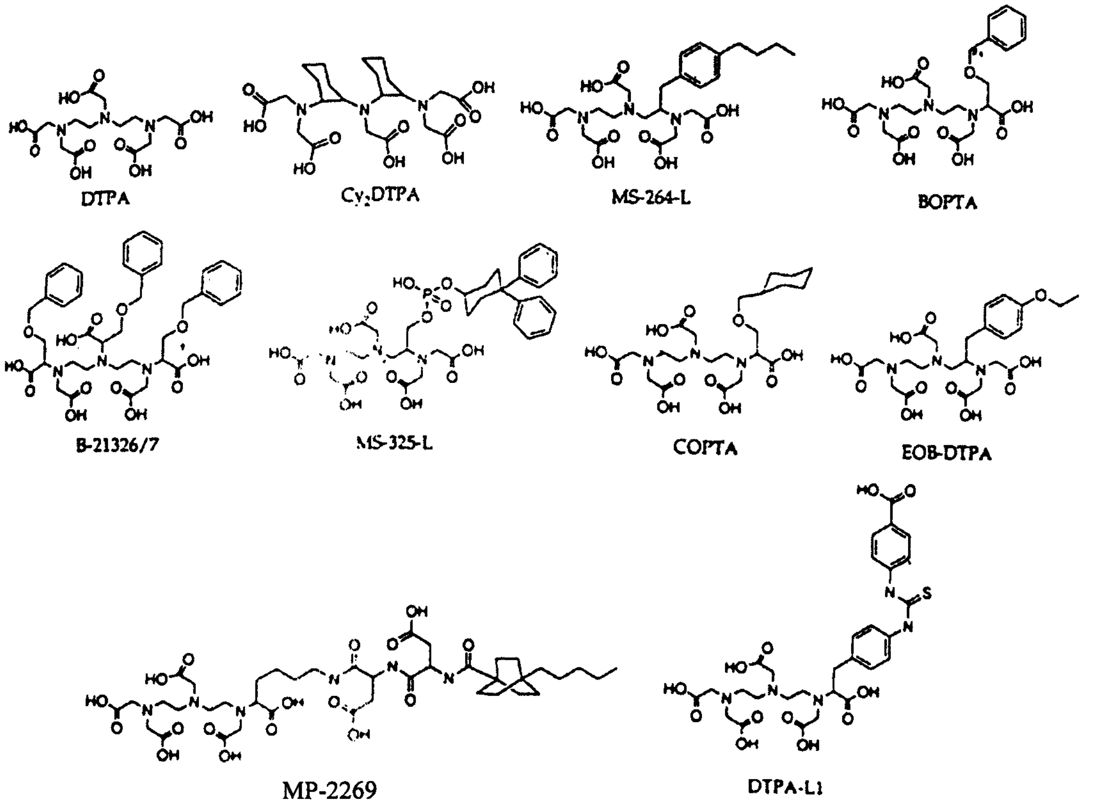

**Fig. 6:** Structures for lanthanide complexes.

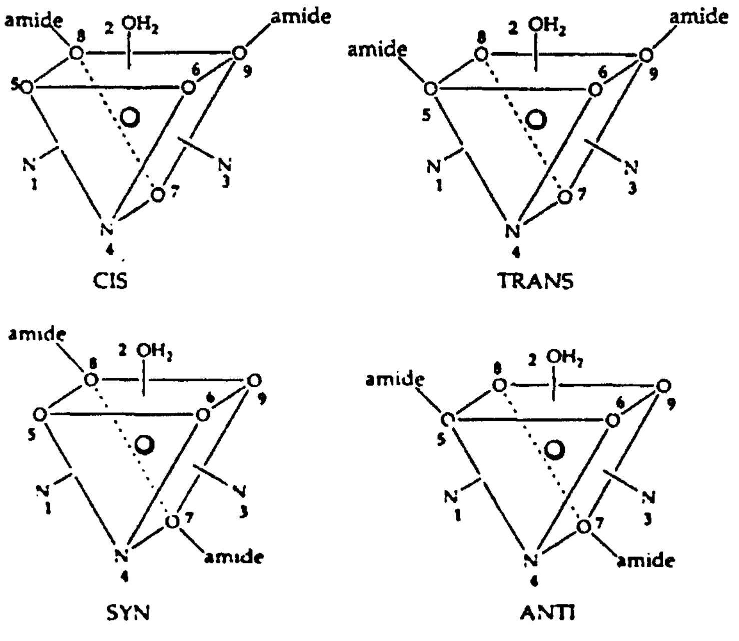

**Fig. 7:** Four possible configurations (cis, trans, syn, and anti) of DTPA bisamides in a TTP arrangement.

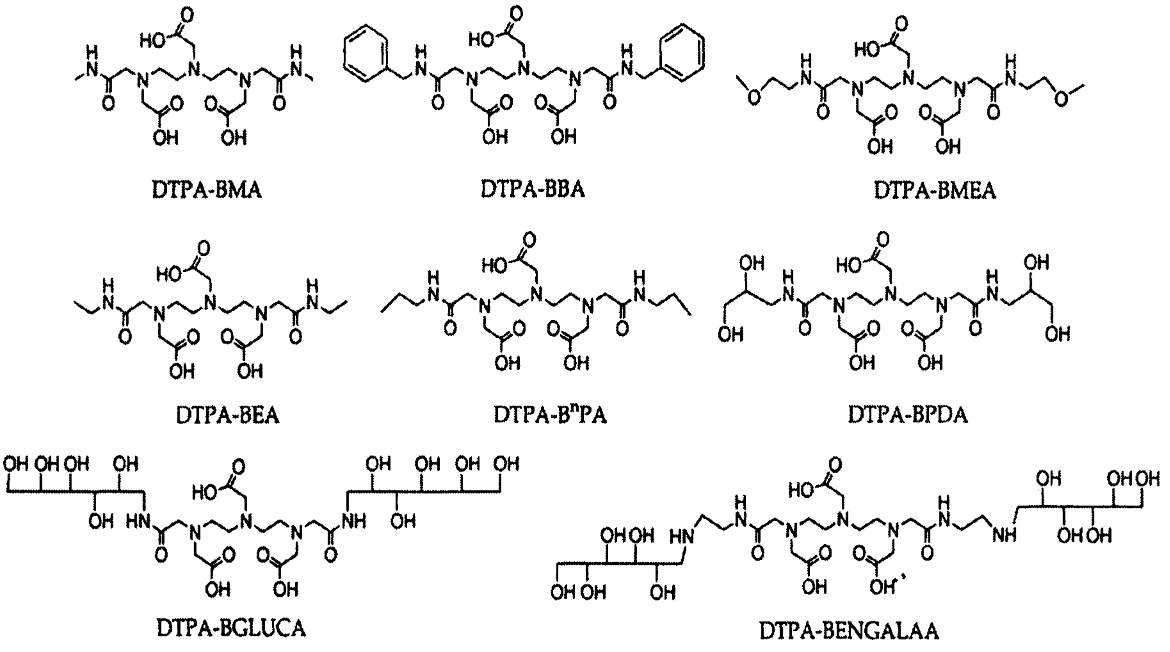

**Fig. 8:** Several structures of DTPA-bisamides.

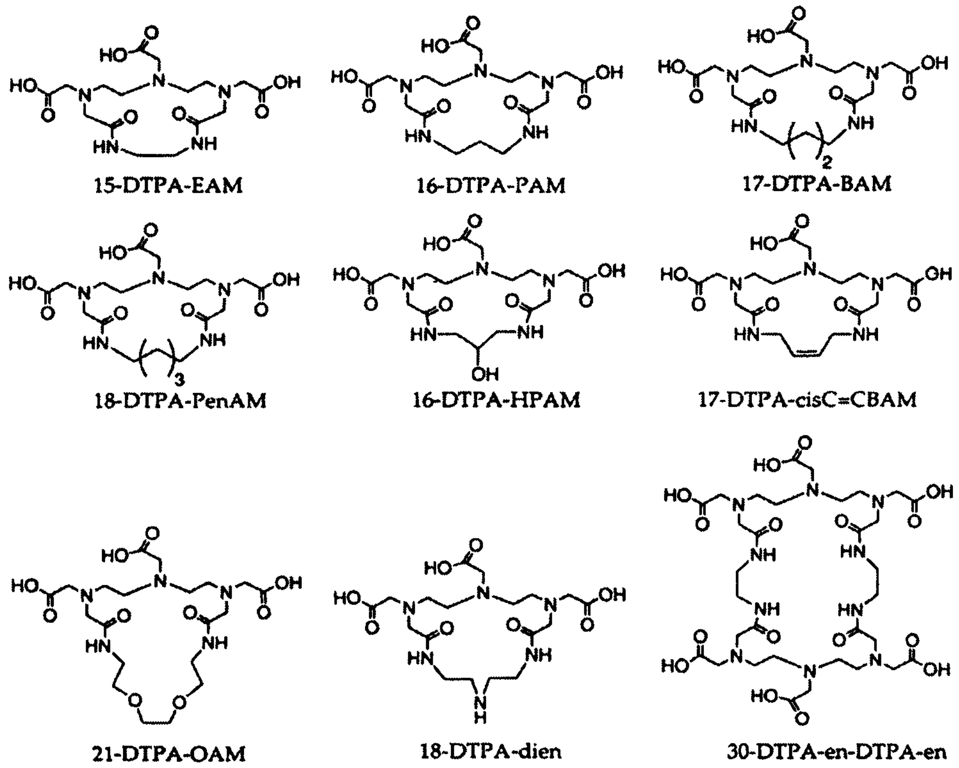

**Fig. 9:** Structures of ligands wrapable around the metal center.

Ln(III) complexes with 18 DTPA-dien crystallize out as $M_4L_4$ tetramers involving nona coordinated environment around Ln(III). 18 DTPA-dien provides eight donor atom to one Ln(III) and bridging carboxylate donor to an adjascent Ln(III).

Like the modified DTPA chelating agents discussed above the cyclic polyamino polycarboxylic acids like DOTA, DOTA (with cyclen units), Fig. 10, have also been modified to form complexes with paramagnetic Ln(III) like Gd(III).

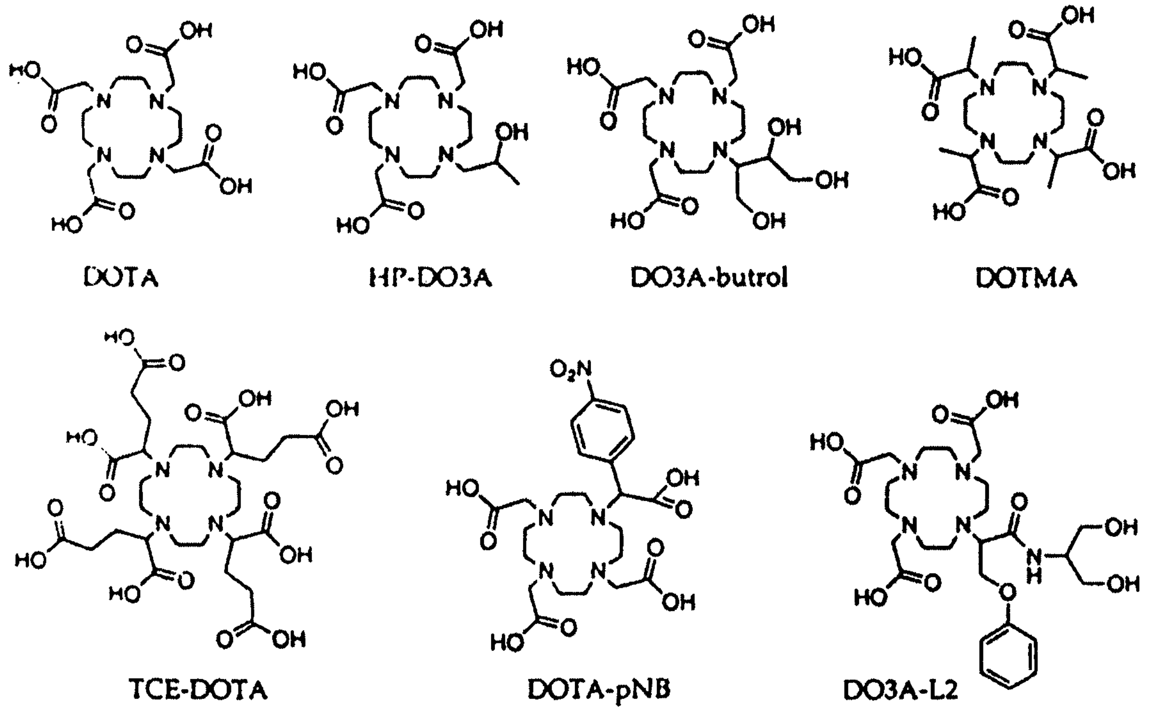

**Fig. 10:** Structures of polyamino polycarbocylic acids with cyclen units.

Rapid exchange interconversion (Fig. 11) between wrapping isomers of Ln(DTPA) was observed to result in a psuedo mirror plane, reducing the number of observed proton resonances by half.

Ln DOTA complexes give solution $^1H$ NMR spectra, which also depict the presence of two interconverting diastereomers. The more predominant isomer has CSAP geometry while the minor one has distorted CSAP structure. Thus there are four stereoisomers, two pairs of enantiomers which are interconvertible in solution by either ring inversion (δδδδ) and (λλλλ) isomers or acetate arm rotation (interconversion of Δ and Λ isomers). Either process alone results in exchange between CSAP and twisted CSAP as shown in Fig. 12.

The simple equation which relates the lifetime, chemical shift and relaxation rates of the solvent molecules in the inner sphere to NMR observable is given by /90/:

$$\frac{1}{T_1^{IS}} = \frac{q\ Pm}{T_{1m} + \tau_m}, \qquad (5)$$

where superscript, IS, refers to inner sphere, Pm is mole fraction of the bound solvent nuclei, q is the number of bound water (or solvent) nuclei per metal ion (i.e., hydration number), $\tau_m$ is the life time of the solvent molecule in the complex and is the reciprocal of the solvent exchange rate $k_{ex}$. The subscript, m, refers to the shift or relaxation rate of the solvent molecule in the inner sphere.

From the above equation one can easily infer that if the water exchange rate is fast enough such that $\tau_m << T_{1m}$, then the relaxation rate enhancement for the coordinated solvent molecule is $(1/T_{1m})$. The above equation clearly demonstrates that increasing the hydration number q, will increase the inner sphere relaxivity. However the increase in q often leads to a decrease in thermodynamic stability as well as kinetic inertness. NMRD (nuclear magnetic relaxation dispersion) or the measurement of relaxation rates as a function of magnetic field, is widely used for characterizing contrast agent /90/. Electronic relaxation parameters are often estimated using NMRD curves to SBM (Solomon-Bioembergen-Morgan) equation.

Coordinatively saturated Gd(III) complexes also enhance relaxivity which occurs via outer sphere relaxation and second sphere relaxation as shown in Fig. 13.

Second sphere relaxation occurs when water molecules in the second coordination sphere (H-bonded to lone pairs on carboxylate oxygen atoms), are relaxed via a dipolar mechanism. Outer sphere relaxation arises from the translational diffusion of water molecules near the Gd(III) complex /103-105/. The water exchange rates for Gd(III) chelate (CA) has been found to be significantly lower (3 to 4) orders of magnitude than that shown by $[Gd(H_2O)_8]^{3+}$. The water exchange rate also varies from one contrast agent to another /106-107/.

Merbach and coworkers /8/ have investigated the contrast agents which are binuclear and trinuclear or even multinuclear chelates (Fig. 14).

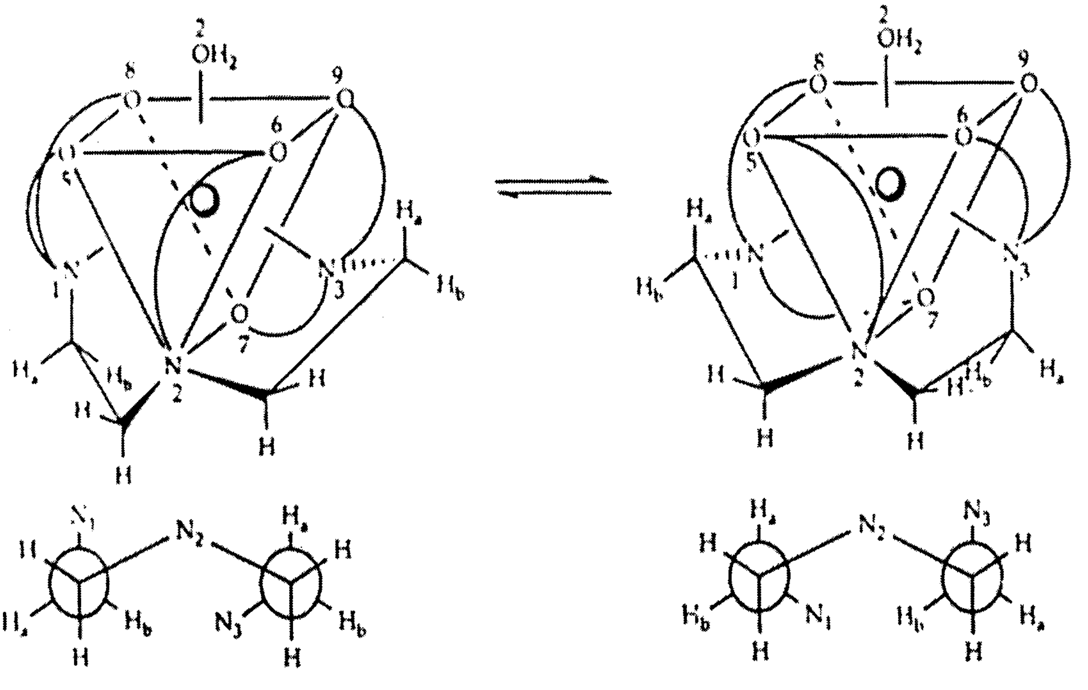

**Fig. 11:** Rapid exchange interconversion between wrapping isomers of Ln(DTPA).

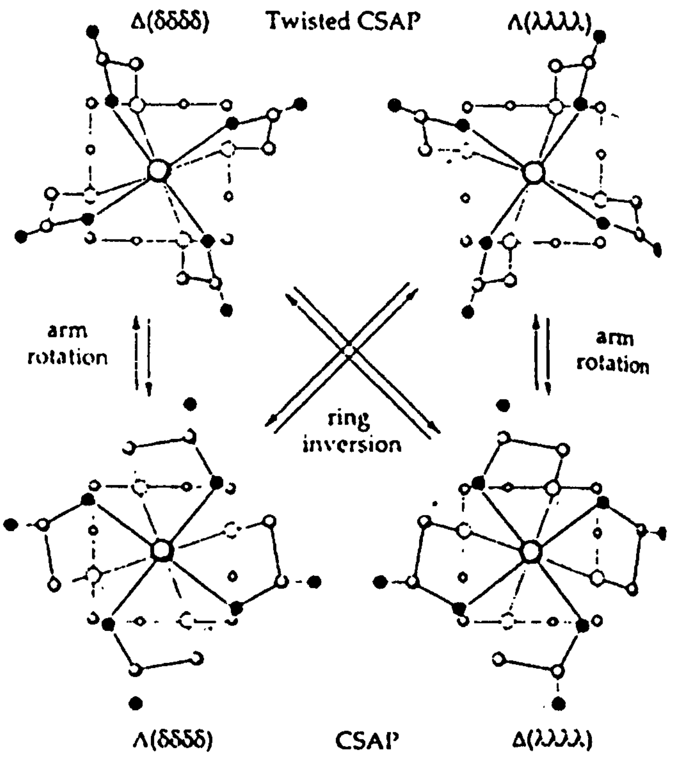

**Fig. 12:** Two diastereomeric conformations (CSAP and twisted CSAP) for lanthanide complexes of DOTA. Pairs of enantiomers exist for the major isomer (Δ-λλλλ) and Λ(δδδδ)) and for the minor isomer (Δ(λλλλ) and Λ (δδδδ)) of $[Ln(DOTA)]^{-}$.

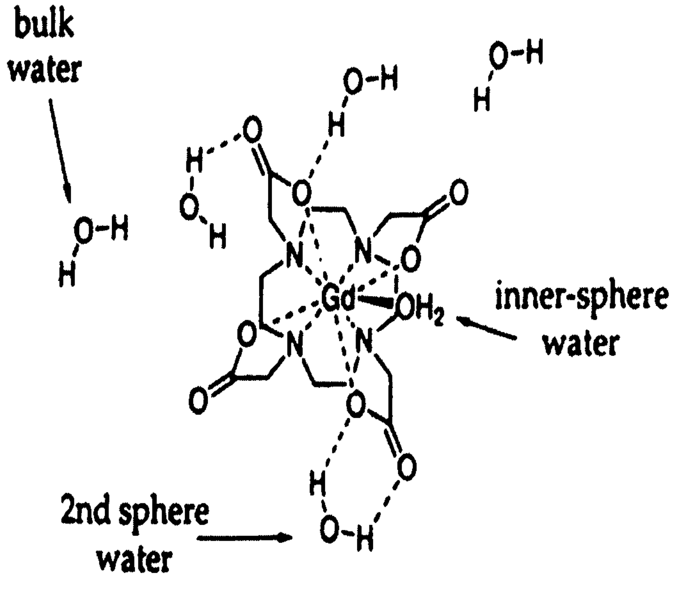

**Fig. 13:** Inner- sphere, 2nd sphere and bulk type water.

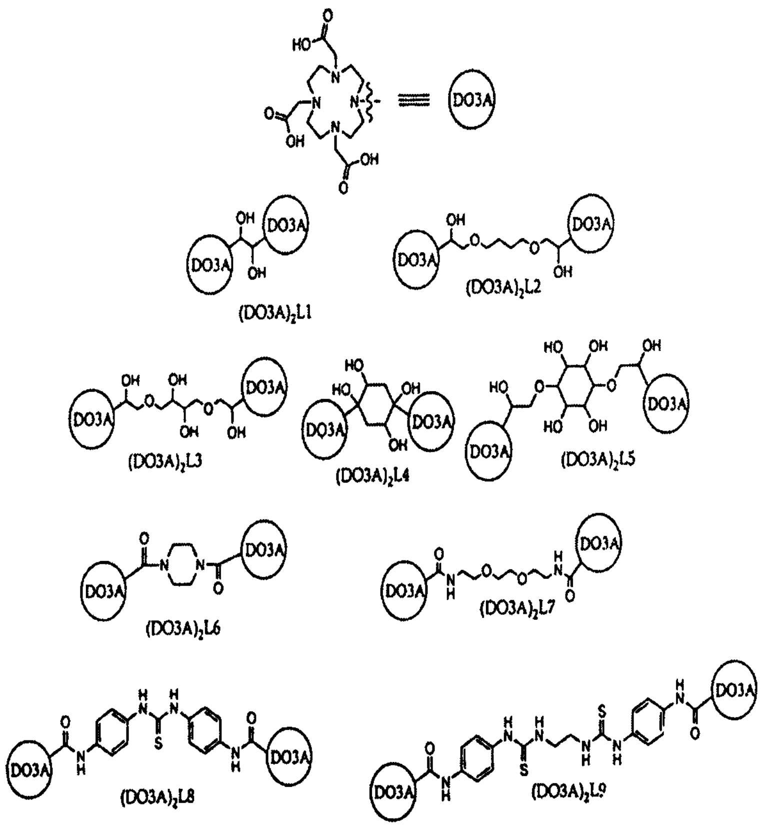

**Fig. 14:** Bi, tri and multi nuclear chelates.

There is a need to improve the relaxivity of the chelates and the obvious way to ameliorate relaxivity is to increase τR. Rigidity obviously causes optimization of $T_R$ as a function of molecular weight, in lanthanide complexes derived from polyamino polycarboxylic acids. Hence a lot of interest has been gathered on conjugation of low molecular weight chelates GdDTPA, GdDOTA and GdHP-DO3A with macromolecular ligands in order to alter remarkably the biophysical and pharmacological properties of the low molecular weight contrast agents. This conjugation increases rotational correlation time and hence the relaxivity in terms of per Gd(III) ion increases significantly. Combined with tissue specific targeting moieties, such polymeric conjugates provide better (Fig. 15) MRI applicability by imaging even the low concentration receptors. Synthetic linear polymers like commercially available polylysines with reactive epsilon amino group of lysine backbone are modified on conjugation with linear and cyclic polyamino polycarboxylates /108/. Polyethelene glycol PEG is another macromolecule which modulates the pharmaco-kinetic properties CA /109/.

Dendrimer based agents of three dimensional oligomers possess highly branched structure e.g. polyamidoamine (PAMAM) (Fig. 16) with uniform surface chemistry. These dendrimers have sufficiently rigid structure and their rolational correlation time is the result of overall tumbling of the molecule. One such commercially available dendrimer is Gadomer – 17 which is derived from a lysine-functionalized 1, 3, 5 benzene tricarboxylic acids /110,111/. GdDTPA-cascade-24-polymer is a PAMAM dendrimer functionalized with 24 Gd-DTPA chelates /112-114/.

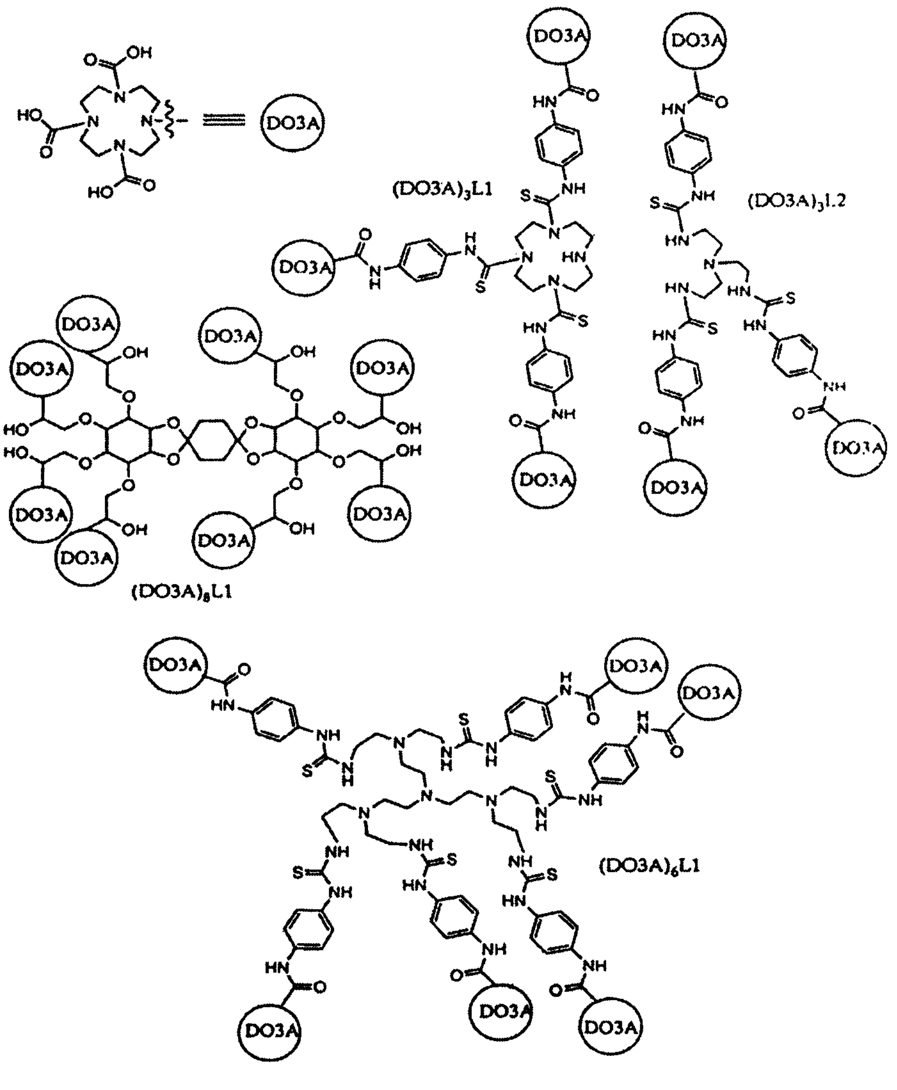

**Fig. 15:** Synthetic linear polymers.

Conjugation of antibodies and other tissue specific molecules to Gd(III) chelates has led to a new type of disease specific MRI contrast agents. Curtet *et al.* found the conjugation of Gd(III)-polylysine chelate with anticarcino embryonic antigen monoclonal antibodies as new contrast agent for MRI /115/.

These conjugated MRI contrast agents with improved relaxivity, function in the body similar to drugs. Their *in vivo* properties like pharmacokinetics, biodistribution safety, etc. are dependent on the properties which determine drug behaviour. The induced hydrophobicity of different degrees, in this structurally different but functionally quite similar new generation of contrast agents, is responsible for their *in vivo* characteristics.

There are several features of CA assisted MRI which make it particularly effective for musculoskeltal and cerebrospinal imaging.

a) superior soft tissue contrast resolution of MRI relative to that of computed tomography(CT),

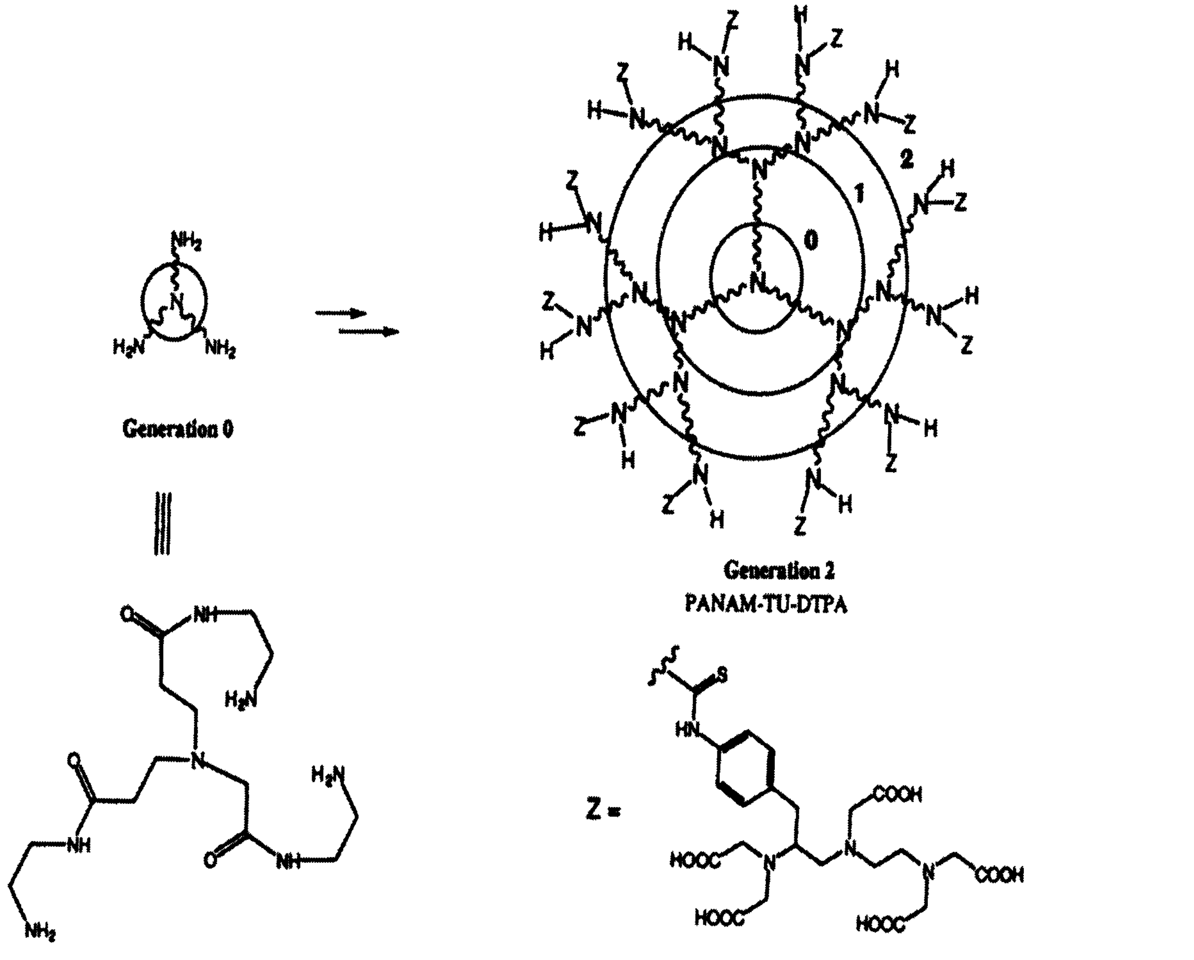

**Fig. 16:** Generation 2 PAMAM thiourea DTPA dendrimer.

b) the ability of MRI to image body directly in the sagittal and coronal planes as well as the axial plane,
c) the ability to vary the level of contrast between the tissues by manipulating the MRI pulse sequence parameters,
d) the lack of artifacts from beam hardening effects, and
e) the capacity to image in presence of metallic hardware (specially effective for musculoskettal imaging)

MRI of the musculoskeletal system currently appears to hold the greatest promise in five major areas.

1) the noninvasive imaging of spine and disc diseases,
2) the early detection of osteonecrosis of the femoral head,
3) the evaluation of the extent and tissue characteristics of musculoskeltal tumors,
4) the assessment of focal and diffuse marrow replacing processes, and
5) the depiction of articular and periarticular structures out of several imaging techniques, the spin echo (SE) technique being most handy and prevalent worldwide. This technique yields gray scale anatomical images in which the intensity of a tissue is a complex function of $T_1$, $T_2$ and spin density of that tissue.

**The relative magnetic resonance Gray scale for body tissue in general is shown in Table 3. By varying the pulse sequence, no doubt one can markedly vary the contrast between two tissues.**

The brighter the image (higher proton density), the shorter is the $T_1$ and/or the longer is the $T_2$. The darker the image (lower proton density), the longer is the $T_1$ and/or the shorter is the $T_2$.

**Table 3**

Magnetic resonance spin-echo gray scale in descending order of brightness

| |
|---|
| Fat |
| Marrow and cancellous bone |
| Brain and spinal cord |
| Viscera |
| Muscle |
| Fluid filled cavities |
| Ligaments and tendons |
| Blood vessels with rapid flow |
| Compact bone |
| Air |

### 8. SOME EXAMPLES USING GADOLINUM(III) DTPA AS CONTRAST AGENTS IN CLINICAL DIAGNOSTIC INVESTIGATIONS INVOLVING CEREBROSPINAL DISEASES AND IN EVALUATION OF CENTRAL NERVOUS SYSTEM

At Vanderbilt, a good number of patients with intracranial neoplastic disease were studied on MRI, both prior to and following intravenous injection of 0.1 mmol/kg of Gd-DTPA in phase I, phase II and phase III clinical investigations. Extensive clinical and laboratory testing were performed before and after CA injection. Serum iron level showed upsurge transiently following injection. However, this serum iron level later on (after 10-40 hrs) had begun to return towards normal and perhaps most noticeable was the total absence of nausea or vomiting associated with the injection of the contrast media. The doses required for required contrast enhancement were about twenty fold lower dose of Gd-DTPA when compared to conventional X-ray contrast agent. No statistical ECG or EEG changes were obtained. Ring enhancement was observed in large tumors with a necrotic centre. Meningiomas typically demonstrated excellent enhancement, classically homogeneous throughout the lesion.

In screening for extra-axial tumors, the use of GdDTPA or other Gd(III) based contrast agent is mandated. Extra-axial tumors, of which meningiomas and acoustic neuromas are common examples, display contrast enhancement on the basis of vascularity. These contrast agents are very useful for other neural origin tumors by remarkably increasing conspicuity. Improved depiction of intra-axial neoplasia with Gd contrast agents, relies mainly upon identification of blood-brain-barrier (BBB) disruption. With primary tumors of glial origin the extent of BBB (blood-brain-barrier) disruption is clearly identified on MRI. Tumor cells are commonly demonstrated on pathology beyond the border of contrast enhancement. However in both children and adults the contrast administration provides better identification of bulk of lesion and thus can serve as a guide for stereotaxic biopsy. Enhancement patterns remarkably improved diagnostic specificity.

When compared with computed tomography, the ability to depict contrast enhancement is far superior on MR. Enhancement on CT is due to increased attenuation of the x-ray beam, and thus only depends on concentration of the reagent. With MR, enhancement depends both on concentration of the paramagnetic CA and on intrinsic relaxation properties of the tissue. The influence of tissue parameters on enhancement together with the absence of beam-hardening artifacts presumably account for superiority of MR over CT in the depiction of contrast enhancement.

### 9. CHELATED Ln(III) COMPLEXES SHIFT-REAGENTS AIDED $^{23}$Na NMR SPECTROSCOPY IN CELLULAR, TISSUE AND WHOLE-ORGAN SYSTEMS

Non destructive observation of intracellular sodium ($Na_i$) levels is of the utmost clinical and biochemical importance. In viable cells, a 10- to 20-fold concentration gradient between $Na_i$ (intracellular sodium ion) and extracellular sodium ion ($Na_o$) is mentioned. Deviation from this gradient is an important indication of detrimental processes. There it becomes a valuable parameter for assessment of cell viability and reversibility of normal function following damage, e.g., an ischemic insult.

Via the various sodium transport mechanisms ($Na^+$-$H^+$ antiport, $Na^+$-$Ca^{++}$ exchange, $Na^+$-$K^+$ ATPase and others), sodium levels and fluxes have an important role in the regulation of intracellular calcium levels, and therefore of cellular function. In muscle cells, e.g. in the myocardium, changes in intracellular sodium ion levels are coupled with contractile function. Real-time, nondestructive monitoring of $Na_i$, therefore, proves a powerful research tool enhancing our biochemical understanding of cellular processes in a healthy and diseased person. Since the methodology of this technique appears to be quite safe, its clinical use definitely would offer *in vivo* markers of localized variation in tissue viability.

$^{23}$Na is a relatively sensitive (much more than $^{31}$P) NMR-observable nuclide, and therefore the intrinsic noninvasive character of NMR could be utilized to achieve the goals described above. Unfortunately, however, the frequency positions (chemical shifts) of intra-and extracellular Na are identical. Together with the fact that total tissue quantity of $Na_o$ is much larger than $Na_i$, this precluded NMR observation of $Na_i$ by NMR. Elgavish and Degani in 1978 /116/ found anionic chelates of paramagnetic Gd(III) as relaxation reagents and could differentiate the Na levels from two compartments. Gupta and Gupta /117,118/ and Pike- and Sprniger in 1982 /119/ introduced paramagnetic Dy(III) complexes as shift reagents for metal cations. Combined with the intrinsic features of NMR spectroscopy, the ability of Ln(III) chelate informs about an aqueous shift reagent, to differentiate the signals from intracellular and extracellular spaces which provided a nondestructive, continuous method to monitor intracellular $Na^+$ ion concentration /117-124/.

$^{23}$Na is a quadrupolar nuclide (spin quantum number, I=3/2) with, natural isotopic abundance of 100%,

NMR receptivity of 9.27 relative to 100 for $^1H$, and average concentration of 44mM in the human body. For nuclide with $I \geq 1$, more than one allowed NMR transition exists. $^{23}Na$, possessing I=3/2 spin, has three transitions, a centre line reflecting the transition between energy levels m = ±1/2 and two satellite lines for transitions m = 3/2 to m = 1/2 and m = -1/2 to m = -3/2. In the absence of any additive effective interaction, the energy gaps give rise to a spectrum of a single resonance with an intensity reflecting the superposition of all three transitions /125/.

In the solid state, the quadrupolar effect lifts the degeneracy among the three transitions and thus splits the spectrum into three resonances and the $T_2$ relaxation is given by equations

$$\left(\frac{1}{T_2}\right)_I = \left[\left(e^2\, q\, Q\right)^2 / 20\right]\left(1+\eta^2/3\right)\tau_c \left[\frac{1}{1\omega_0{}^2\tau_c{}^2} + \frac{1}{1+4\omega_0{}^2\tau_c{}^2}\right] \tag{6}$$

$$\left(\frac{1}{T_2}\right)_{II} = \left[\left(e^2\, q\, Q\right)^2 / 20\right]\left(1+\eta^2/3\right)\tau_c \left[1+\frac{1}{1\omega_0{}^2\tau_c{}^2}\right], \tag{7}$$

where $e^2$ q Q is the magnitude of the nuclear quadrupole interaction (in rad/sec), $\eta$ is dimensionless asymmetry factor defined as (Vxx – Vxy) / $V_2x$, the V components are the second derivatives of the electrical potential and constitute the principal components of electric field gradient, $\tau_c$ is the correlation time and $\omega_0$ is the Larmor frequency. $(1/T_2)_I$ decay represents 40% of the signal and can be attributed to the transition between the +1/2 and –1/2 Zeeman energy levels /126,127/. The remaining 60% decays at the rate $(1/T_2)_{II}$ and can be attributed to 3/2 to 1/2 and –1/2 to –3/2 transitions.

The method of induced differentiation in NMR spectra of aqueous cationic species by paramagnetic lanthanide chelate shift reagents was introduced by Elgavish *et al.* and Gupta in 1982 used shift method to solve the problem of separation of $^{23}Na$ NMR signals $Na_i$ and $Na_o$, in cells and tissues using a Lanthanide chelate shift reagent. These shift reagents do not cross cell membrane and thus, interacting only with extracellular ions, they cause a paramagnetic shift in the frequency position of $Na_o$ signals away from unshifted position of $Na_i$. Thus the discrimination between $^{13}Na$ NMR resonances in two compartments, extracellular and intracellular, is enabled by the fact that the appropriate shift reagents are membrane impermeable, so that only the extracellular resonance is affected by paramagnetic lanthanide chelate.

Three different types of chelating ligands (Fig. 17) which form stable complex with $Dy^{3+}$ have been most extensively used as NMR shift reagents.

Dy(III) chelated to two equivalents of the PPP ligand and gives the largest shift known, but has a tendency to bind $Ca^{++}$. Ln(III) complexes containing PPP can be troublesome to use in buffer solution because they bind $Ca^{++}$ and may precipitate. Some modifications are proposed, which allows the application of $Dy(PPP)_2$ as shift reagent for monitoring compartmentation of Na during onslaught of ischemia by $^{23}Na$ NMR spectroscopy /127/ (Fig. 18).

Despite this disadvantage $Dy(PPP)_2{}^{7-}$ has two major advantages, (i) its large induced shift which produces excellent spectral resolution and (ii) its simple spectrum, i.e., two resonance, a truly unshifted $Na_i$ and an upfield shifted $Na_o$ signal[11].

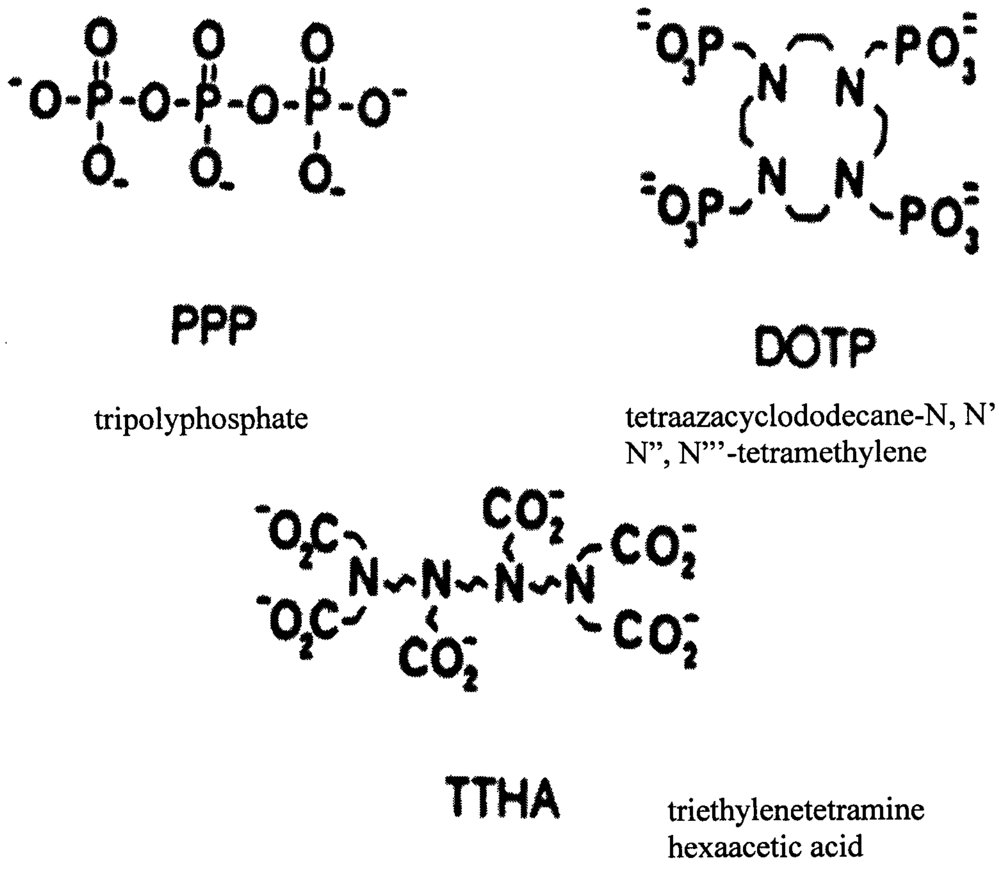

**Fig. 17:** Chemical formula of three shift reagents PPP, DOTP and TTHA.

The shift reagent Dy (TTHA) can tolerate a large concentration of $Ca^{++}$. However, Na NMR spectra in organs in presence of Dy (TTHA) display more signals than with other shift reagents. $Na_0$ resonance spectra of hearts perfused with Dy (TTHA) have seven to eight poorly resolved peaks. Kohler *et al.* performed a series of modeling, ischemia and reflow experiments in perfused hearts of various species. Their work led to better understanding of the reasons for the complexity of Dy (TTHA) aided $^{23}Na$ NMR spectroscopy.

In 1987 Sherry *et al.* /127/ introduced the $Dy^{3+}$ complex of 1,4,7,10-tetraazacyclododecane, 1,4,7,10, tetrakis (methylene phosphonate) DOTP as a SR (shift reagent) and showed that it produced a $^{23}Na$ shift in red cells compared to that induced by $[Dy(PPP)_2]^{-7}$. The primary advantages of this S R are its thermodynamic stability and kinetic inertness in biological tissue and its geometry. Its structure shows that macroscopic DOTP ligand encapsulates a lanthanide(III) ion into a symmetrical structure with four phosphonate side arm chain ligands forming a charged surface for ion pairing with sodium ion. This position for the $Na^+$ binding sites along with 4 fold axis of symmetry of the complex optimum phosphonate surface also provides a strong ion which is about the same as found with $[Dy(PPP)_2]^{7-}$. Nevertheless these complexes are very stable *in vivo* and paramagnetic shifts induced by $Dy(DOTP)^{5-}$are large in comparison to those induced by $Dy(DOTP)^{3-}$ at an equivalent concentration. Thus considerably less S R will be required to separate intra- and extracellular $^{23}Na$ resonances. Since $^{23}Na$ hyperfine shifts induced by $Tm(DOTP)^{5-}$ were

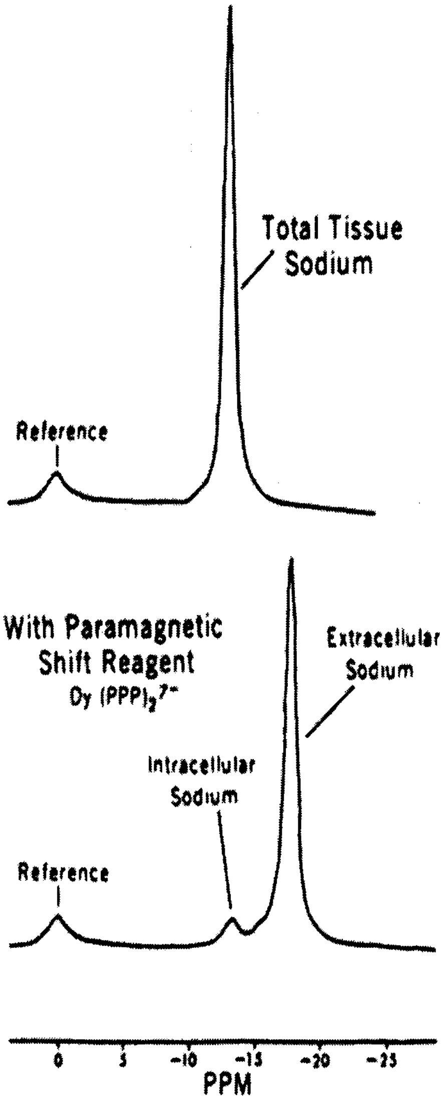

**Fig. 18:** $^{23}Na$ NMR spectra of an isolated perfused rat heart in the absence (top) and presence (bottom) of the paramagnetic shift reagent Dy(PPP)2.

equal to those induced by $Tm(DOTP)^{5-}$, this prompted these workers to try $Tm(DOTP)^{5-}$ as $^{23}Na$ SR which is for superior in a perfused heart preparation than $Dy(DOTP)^{3-}$. The amount of $Tm(DOTP)^{5}$ required for the separation of $Na^{+}_{i}$ and $Na_{0}$ was reported to be 3 fold lower than that typically required by Dy TTHA. This has significant advantages in two respects. First, the $^{23}Na$ line widths are substantially narrower when Tm $DOTP^{5-}$ is used S R, making it easier to separate intra- and extracellular sodium resonances and second, the bulk susceptibility gradient across the sample (i.e. a perfused heart suspended in 18-20 mm M R tube typically has a significant volume of perfusate surrounding the organ) is substantially less with $Tm^{3+}$ reagent, Tm DOTP has been used in a number of investigation /128-133/ for monitoring $Na^{+}_{i}$ and $Na^{+}_{o}$ in isolated, perfused heart. The use of triple quantum filter (TQF) technique could eliminate most of the $Na^{+}_{o}$ resonance from perfused heart preparation by discriminating against $^{23}Na$ undergoing mono exponential relaxation. This reduced intensity of $Na_{0}$ resonance, shifted to the high frequency by Tm $DOTP^{5-}$, so that its intensity becomes comparable to that of $Na^+_i$ resonance in a number of *in vivo* studies.

S R aided $^{23}Na$ and $^{31}PMRS$ has been used by Xia *et al.* to monitor $Na^+_i$, Mg intra free $^{+2}$ signals and intracellular pH in the *in vivo* rat liver after a significant surface burn injury again using Tm $DOTP^{5-}$ to resolve $Na^+_i$ and $Na^+_o$ signals. Such a study could show that even by using a much smaller dose of Tm $DOTP^{5-}$ there was clear cut observed separation of $Na^+_i$ and $Na^+_o$ signals in 24 hrs burn animals compared to control animals. $^{23}Na$ M R S examination of the *in vivo* kidney upon infusion of Tm $DOTP^{5-}$ gave interesting results. After 10 min of Tm $DOTP^{5-}$ infusion to a dose of ~0.15 m moles/kg, one broad resonance was evident near 15 ppm which continued to shift field with time and a second resonance appeared as a high frequency shoulder on the unshifted intracellular resonance after about 15-16 minutes. These three resonances could be resolved nicely while Dy TTHA could not resolve these same resonances in $^{23}Na$ MR spectra of *in vivo* kidney. This was ascribed to two main reasons: First about 3 fold more Dy $TTHA^{3-}$ was required for producing the same peak separation as Tm $DOTP^{5-}$ in *in vivo* spectra and second, the greater bulk magnetic susceptibility of Dy $TTHA^{3-}$ induced greater $T_2$ time broadening effect in $^{23}Na$ resonances. Sherry *et al.* compared these *in vivo* spectra with $^{23}Na$ spectrum of blood and urine samples, collected during infusion. Each S R allowed an assignment of highly shifted $^{23}Na$ resonance only (detected in Tm $DOTP^5$) to filtrate $Na^+$ and less highly shifted $^{23}Na$ resonance to interstial and vascular $Na^+$ /7, 8, 122, 123/.

### 10. APPLICATION OF LANTHANIDE COMPLEXES IN AQUEOUS SOLUTION ESPECIALLY WITH BIOLOGICAL RELEVANCE

There has been much interest in the use of lanthanide complexes as shift reagents (S R) in NMR to effect spectral simplification and resolution enhancement. Lanthanide complexes, especially in solution, continue to be used as paramagnetic probes in biological studies to gain structural information on proteins, nucleotides and amino acids /134-136/. There has been an increasing interest in the application of lanthanide coordination complexes in molecular recognition and chirality sensing of biological substrates. The chirality occupies the nucleus in such studies of molecular basis, biological and artificial chemistry, and is manifested by natural products: proteins, nucleic acids, sugars, polypeptides, hormones and antibiotics and also by drugs, food and other synthetic chemicals. Since these substances often exhibit specific activity and functionality depending on their chirality, methods are being developed for determination of absolute configuration of chiral substrates and separation of their enantiomers. The tris (β - diketonate) of lanthanides in particular ($Pr^{3+}$ and $Eu^{3+}$) exhibited further interesting molecular recognition properties upon highly coordinated complexation with substrates like amino acids, polypeptides and even proteins by forming ternary negatively charged complex with anionic guest /137/ (Fig. 19).

Amino acids and oligopeptides are the most fundamental substrates in biological and artificial processes. When these are targeted, the recognition and sensing require almost neutral pH (pH 6-7.5). Lanthanide tris (β - diketonates) have successfully been used in liquid membrane transport of amino acids /138-139/. Kojuma *et al.* have synthesized two new chiral ligands (Fig. 20 a, b) for Ln(III) which have been found to be very effective in neutral aqueous solutions /140-141/. Kabuto *et al.* reported the effectivity of $Eu^{3+}$ complex of N, N, N', N' tetra kis (2- pyridyl methyl) – (R) - propylene di amine /142/ (Fig. 20 c).

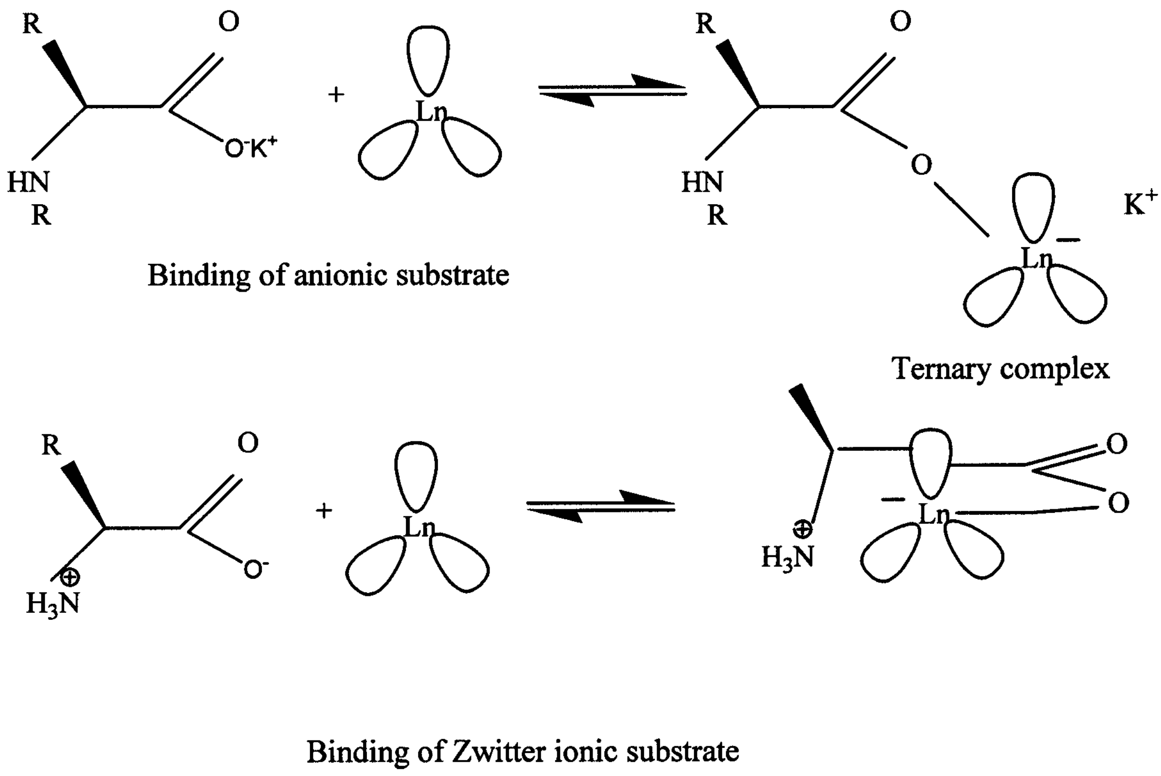

**Fig. 19:** Binding of anionic and Zwitter ionic substrate.

Lanthanide knowledge of the distribution of biologically important anions and cations over intracellular and extracellular compartments is of considerable value in cell biology and of great potential use in medical diagnosis.

### 11. LUMINESCENT Ln(III) COMPLEXES AND THEIR BIOLOGICAL RELEVANCE

Lanthanide chelates specially derived from Eu(III), Tb(III), Yb(III) and Nd(III) give characteristic luminescence spectra, but the associated excited lifetimes extends into micro to millisecond domain which is a crucial advantage for the design of luminescent materials with several practical applications, especially as LUMINESCENT FLAG for

1) labeling biological materials /143-145/
2) sensing nonchiral /135,146/ and chiral analytes /147/, and
3) probing metallic environment

The lanthanides are characterized by sharp parity forbidden bands with $\varepsilon$ = less than 10 $M^{-1}$ $cm^{-1}$. However some bands shown to exhibit high sensitivity and substantial intensification are called hypersensitive bands. These bands obey ($\Delta J$) selection rules. We have shown that under the influence of

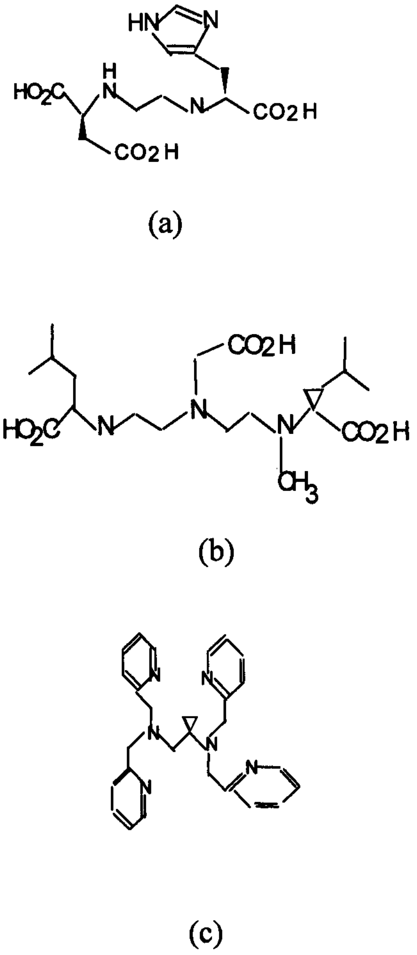

**Fig. 20:** Structures of chiral ligands.

highly chelating ligands, often the f-f bands, which otherwise were considered only negligibly sensitive, also gain substantial sensitivity towards coordination changes and hence termed pseudohypersensitive bands. The variation of intensities of different 4f-4f bands, when there is change in the structure and conformation of the lanthanide-biomolecular complexes, have been found a good way to monitor some isomorphous $Ca^{++}$ substitution from biomolecules by paramagnetic lanthanides /148-150/.

Another important practical consequence of the sharp, forbidden 4f-4f transitions concerns the production of sufficient radiant power per bandwidth compatible with stimulative emission and laser functions. The growing need for efficient laser at wave lengths in the near infrared region has recently motivated new development of Nd(III), Er(III) and Tm(III) garnets /151,152/.

Luminescent laser sensor or probe designing requires a combination of the following factors as needed:

a) protection of included lanthanide ions from quenching by high energy vibrations (solvent molecules or ligating groups)

b) multiple absorbing groups suitable for light harvesting and energy transfer in order to efficiently feed metal centered excited states c) high thermodynamic stability and kinetic inertness, and
d) specific solubility and non toxicity of these sensors are used in biological media and clinical investigations /153,154/.

Prof. Bunzli and Piguet have done extremely innovative and at the same time highly exhaustive work on lanthanide containing polymetallic functional assemblies. The fascinating expansion of lanthanide coordination chemistry and supramolecular chemistry has triggered practical successes in catalysis, biomedical analysis, clinical diagnostics and also some success in therapeutic medicine. The intrinsic chemical (Lewis acidity), magnetic and spectroscopic, mainly luminescence and to some extent 4f-4f transition spectral properties of these Ln(III) ions, often not matched by those of other transition metal ions, make lanthanides privileged partner for the design of functionalized molecular species and materials especially when lanthanides are easily available commercially in highly pure form.

### 12. THE PERSPECTIVE FOR INTEGRATED PHARMACOLOGICAL ACTIVITIES OF LANTHANIDES

The cells respond to the attacking metal ions as a multiple target system, in which various reactions with various targets are organized to a sequence of events. The ultimate biological effect is actually the integrated effect of these events. Ji *et al.* /155/ have given a nice account of the events that happen when Ln(III) ions attack a cancer cell and induce apoptosis, and this they consider as the core of the lanthanide potential as anticancer activity. Along with apoptosis, there are several synergic related effects, ROS scavenging, cell protection, cytoskeleton stabilization and also immunologic enhancement. They proposed that lanthanides role in affecting cancer involve the integrated synergic effect, as shown in Fig. 21.

However, the above scheme has not been supported by experimental evidence and hence it is risky to draw any clear cut conclusions. No doubt the significant accumulation of Ln(III) selectivity in tumor tissue is well documented but it does not mean that they can selectively affect cancer cells. Nie *et al.* /156/, Ji *et al.* /155/, and Li *et al.*/157/ observed that with different cell lines, an appropriate dose may inhibit substantially the cancer cells without significant influence on the normal cells.

$CeCl_3$ and $NdCl_3$ incubation decreased the level of CaM (Calmodulin) level in cancer cell K562, but increased that in normal human cells /155/. These workers could not conclusively suggest any selectivity of Ln(III) for cancer cells at the same time they did not rule out any possibility of the selectivity of Ln for cancer cells.

### ACKNOWLEDGEMENT

SNM thanks the Department of Science and Technology, New Delhi for a research grant and Dr. P. K. Ghosh, Director and Prof. B. N. Jha, Discipline Coordinator of Marine Algae & Marine Environment Discipline, Central Salt and Marine Chemicals Research Institute, Bhavnagar for their encouragement.

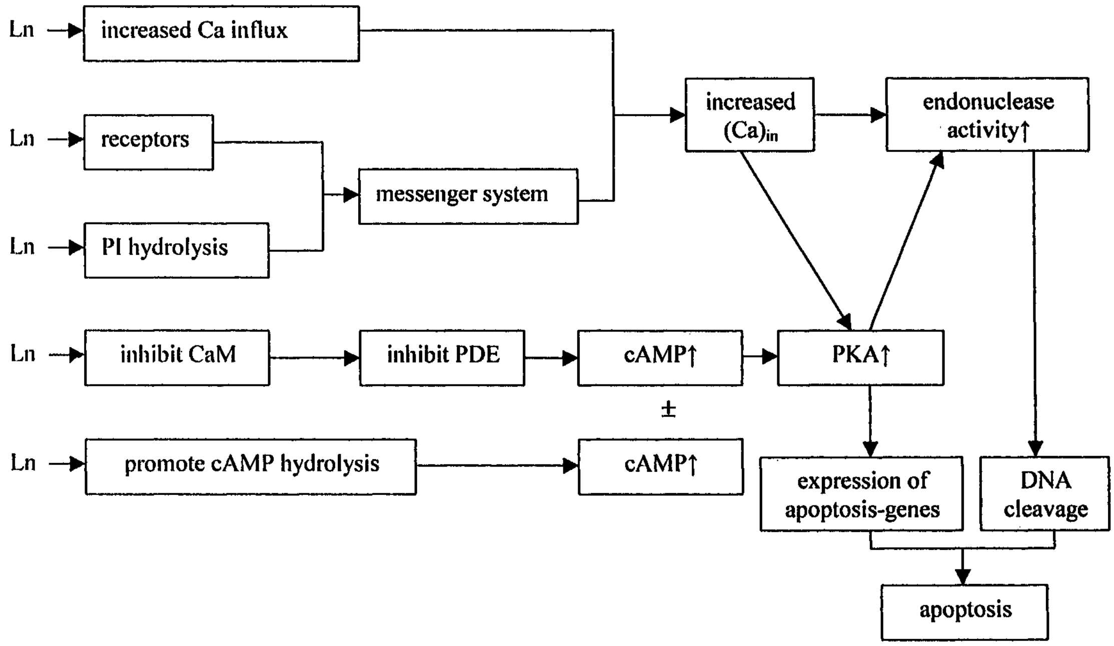

**Fig. 21:** The possible pathways leading to Ln induced apoptosis.

### REFERENCES

1. C. H. Evans, "Biochemistry of Lanthanides", Plenum Press, New York, Chapter 1, 2 (1990).
2. P. O. Webster, Akt. Atom Stockholm A E, ISS PP, (1965).
3. M. Esposito, M. Oddone, S. Accardo and M. Cutoto, *Clin Chem.*, **32,** 1398 (1986).
4. M. Esposito, P. Collechi, S. Brera, E. Mor, A. Mazzucotelli, M. Cutoto and M. Oddone, *Sci. Total Environment,* **50,** 55 (1986).
5. E. Sabbioni, L. Goetz, G. Bignoli, Assessment of European Communities Situation Report, Eur 698/IV, (1982).
6. E. Sabbioni, R. Pietra, P. Gaglione, G. Vocaturo, P. Colombo, M. Zanoni and F. Rodi, *Sci. Total Environment,* **26,** 19 (1982).
7. P. Caravan, J. J. Ellison, T. J. McMurry, R. B. Lauffer, *Chem. Rev.*, **99,** 2293 (1999).
8. "The chemistry of contrast agents in medical magnetic resonance imaging", A. E. Merbach and E. Toth (Eds.), Wiley, New York (2001).
9. J. A. Peters, J. Huskens, D. J. Raber, *Prog. Nucl. Magn. Reson. Spectrosc.* **28,** 283 (1996).
10. M. Komiyana, N. Takeda, H. Shigekawa, *J. Chem. Soc. Chem. Commun.*, 1443 (1999).
11. I. K. Hemmila, Applications of Fluorescence in Immunoassays, Wiley and Sons, New York (1991).
12. G. Mathis, *Clin. Chem.*, **41,** 1391 (1995).
13. I. K. Hemmila, T. Stahlberg, P. Mottvam, "Bioanalytical Applications of Labeling Technologies", Wallac Oy., Turka (1995).
14. G. Mathis, In: "Rare Earths" , R. Saez-Puche and P. Caro (Eds.), Editorial Complutence, S A, Madrid 285 (1998).
15. J. C. G. Bunzli and C. Piguet, *Chem. Rev.*, **102,** 1897 (2002).

16. D. Parker, *Coord. Chem. Rev.*, **205**, 109 (2000).
17. J. C. G. Bunzli and C. Piguet, *Chem. Soc. Rev.*, **28,** 347 (1999).
18. C. Gorller-Walrand and K. Binnemans, In: "Handbook on physics and chemistry of rare earths", Amsterdam, Elsevier, K. A. Gschneidner and L. Eying (Eds.), Vol. 25 (1998).
19. S. N. Misra and S. O. Sommerer, *Appl. Spectrosc. Rev.*, **26,** 152 (1991).
20. S. N. Misra and K. John, *Appl. Spectrosc. Rev.*, **28** 285 (1993).
21. S. N. Misra and S. O. Sommerer, *Rev. Inorg. Chem.*, **12,** 157 (1992).
22. S. N. Misra, G. Ramchandriah, M. A. Gagnani, M. Indiradevi and R. S. Shukla, *Appl. Spectrosc. Rev.*, **38**, 433 (2003).
23. E. Toth, L. Burai and A. E. Merbach, *Coord. Chem. Rev.*, **216,** 363 (2001).
24. S. Aime, M. Botta, M. Fasano and E. Terrano, *Chem. Soc. Rev.*, **27**, 19 (1998).
25. E. Terrano, S. Aime, S. Barge, D. D. Castelli and F. U. Niesen, *J. Inorg. Biochem.*, **86,** 452 (2001).
26. C. F. G. C. Gereldes, In: NMR in "Supramolecular Chemistry", M. Pans, (Ed.), Kluwer Press, Netherland 133 (1999).
27. A. Bianchi, L. Calabi, F. Corona, S. Fontana, P. Losi, A. Maiocchi, L. Paleari and B. Valtancoli, *Coord. Chem. Rev.*, **204,** 309 (2000).
28. T. D. Mody and J. L. Sessler, In: "Supramolecular Technology", D. N. Reinhoudt (Ed.), John Wiley, New York, Chapter 7, 245 (1999).
29. T. D. Mody and J. L. Sessler, *J. Porphyrin Pathalocyanines*, **5**, 134 (2001).
30. F. H. Allen and O. Kennard, *Cambridge Structural Database,* October (2000).
31. N. Kaltsoyannis and P. Scott, "The f-Elements" of Oxford University Press, New York (1999).
32. J. C. G. Bunzli, In: "Rare Earths", R. Saez-Puche and P. Caro (Eds.), Editorial Complutence, S A, Madrid 223 (1998).
33. S. N. Misra, *Indian J. Chem.*, **35B**, 761 (1997).
34. S. N. Misra, "Lanthanides: Chemical, Biochemical and Medical Curiosities", Monograph Published by Bhavnagar University, UGC Project, October (1998).
35. E. El-Fakahany, J. Lopez and E. Richelson, *J. Neurochem.*, **40,** 1687 (1983).
36. S. N. Cohen, C. T. Chang and L. Msu, *Proc. Natl. Acad. Sci.*, **69,** 2110 (1972).
37. M. Mandel and A. Higa, *J. Molec. Biol.*, **53,** 159 (1970).
38. J. Z. Ni, D. Q. Zhao, X. M. Li, In: "Ten Years Progress in Bioinorganic Chemistry in China", K. Wang and W. S. Han (Eds.), Higher Education Press, Beijing 19 (1997).
39. J. Z. Ni, "Bioinorganic Chemistry of Lanthanides", Science Press, Beijing (1995).
40. K. Wang, R. Li, Y. Cheng and V. Zhu, *Coord. Chem. Rev.*, **297,** 190 (1999).
41. K. Wang, *S. Afr. J. Chem.*, **50,** 232 (1997).
42. D. Y. Huang, S. J. Wu, D. L. He, Z. F. Luo and L. Shi, *Chinese Biochem. J.*, 231 (1998), Special issue of 8th National symposium on Biochemistry and Molecular Biology.
43. a. R G. Canada, *Biochem. Biophys. Res. Commun.*, **111,** 135 (1983). b. R. G. Canada, P. A. Andrews, K. M. Mack and A. Haider, *Biochim. Biophys. Acta.*, **25,** 1267 (1995).
44. X. D. Yang, X. T. Liu, B. W. Chen, R. C. Li and K. Wang, In: "Proceedings of First SinoDutch

Workshop on the environmental behaviour and ecology of rare earth", R. Bosman, J. L. M. de Boer and P. M. van Berkel (Eds.), TNO-MEP, Netherland 205 (1997).

45. Y. Cheng, C. L. Bai and K. Wang, *Biochem. Biophys. Acta.*, (2002) in press.
46. X. M. Li, Y. F. Zhang, J. Z. Ni, J. W. Chen and F. H. Wang, *J. Inorg. Biochem.*, **53,** 73 (1994).
47. F. Hwang, D. Zhao, J. Chen, X. Chen and J. Z. Ni, *Chem. Phys. Lipids*, **82,** 73 (1996).
48. J. Seelig, R. Lehrmann and E. Terzi, *Mol. Membr. Biol.*, **12,** 51 (1995).
49. W. D. Wu, X. F. Qin and O. G. Jiang, *J. Hug. Toxicol.*, **8,** 201 (1994).
50. B. Xiao, J Y J "Rare earths bioinorganic chemistry", "In Reports on Basic Research on Lanthanides" Item 8, State Commission of Science and Technology", Beijing, China 121 (1996).
51. P. Soto, H. Rodrigues and O. Monasterio, *Biochemistry*, **35,** 6337 (1996).
52. S. N. Misra, G. Joseph, K. Anjaiah, H. C. Bajaj and K. Venkatsubramanian, *Indian J Chem.*, **29A,** 346 (1990); S. N. Misra, G. Joseph, K. Anjaiah and S. H. R. Abdi, *Indian J. Biochem. Biophys.*, **29,** 70 (1992).
53. S. N. Misra, *Indian J. Biochem. Biophys.*, **27,** 284 (1990); S. N. Misra and S. B. Mehta, *Bull. Chem. Soc.*, Japan **64,** 3653 (1991).
54. G. Joseph, Ph. D. Thesis, Bhavnagar University, Bhavnagar – 364 002 (1988).
55. K. Anjaiah, Ph. D. Thesis, Bhavnagar University, Bhavnagar – 364 002 (1990).
56. S. J. Shah, Ph. D. Thesis, Bhavnagar University, Bhavnagar – 364 002 (1992).
57. M. J. Shah, Ph. D. Thesis, Bhavnagar University, Bhavnagar – 364 002 (1992).
58. C. M. Suveerkumar, Ph. D. Thesis, Bhavnagar University, Bhavnagar – 364 002 (1997).
59. L. C. Maxwell, F. Bischoff and E. M. Ottrey, *J. Pharmacol. Exp. Ther.*, **43,** 61 (1931).
60. L. J. Anghileri, *Eur. J. Cancer*, **15,** 1459 (1979).
61. L. J. Anghileri, C. Marchal, M. C. Crone-Escanye and J. Roberts, *Tumor*, **71,** 39 (1985).
62. L. J. Anghileri, M. C. Crone-Escanye and J. Roberts, *Gesch. Wuls. Forsch.*, **53,** 338 (1983).
63. L. J. Anghileri, *Recent Results Cancer Research*, **109,** 106 (1988).
64. S. N. Misra, *Indian J. Chem.*, **35B,** 761 (1996).
65. S. N. Misra and S. Jain, Unpublished results Preliminary Findings.
66. S. N. Misra, T.N. Mishra and R.C. Mehrotra, *Indian J. Chem.*, **5,** 372 (1967).
67. Editorial Staff, Diagnostic Imaging Industry Report, update (1996); Miller Freeman, San Franciecs, (1996).
68. P O V Reports, "The Diagnostic Imaging", Marketplace in USA, POV Inc, Cedar Grave, New Jersey (1998).
69. J. Jeans and Vermeeren, *Eur. J. Nucl. Med.*, **25,** 1469 (1998).
70. S. B. Yu and A. D. Watson, *Chem. Rev.*, **99,** 2353 (1999).
71. S. E. Seltzer, *Invest Radiol.*, **14,** 356 (1979); *J. Comput. Assiot. Tomogr.*, **5,** 370 (1981).
72. T. S. Curry, J. E. Downdey, R. Mury, In: CHRISTENSENS physics of Diagnostic Radiology", 4th edition, Lea Febiger, Philadelphia, USA (1992).
73. W. R. Hendec and R. Ritenour, "Medical Imaging Physics", Mosby- year Book, St. Louis, 3rd edition (1992).

74. E. A. Janon, *Am. J. Roentgen*, **152,** 1348 (1989).
75. W. H. Buch and D. P. Swanson, *Am. J. Roentgen*, **157**, 1153 (1991).
76. R. J. Gibson, C. I. Meanock, E. P. H. Torre and T. M. Walker, *Clin. Radiol.*, **47,** 278 (1993).
77. A. D. Quinn, N. J. O'Hare, F. H. Wallis and G. F. J. Wilson, *Comput. Assist. Tomogr.*, **18**, 634 (1994).
78. M. R. Rudnick, S. Goldfarb, L. Wexler, P. A. Lud brook, M. J. Murphy, E. F. Halpern, H. A. Hill, M. Winnifork, M. P. Cohen and D. B. Van Fossen, *Kidney Int.*, **47**, 254 (1995).
79. Y. Kinno, K. Odagiri, K. Andoh, Y. Itoh and K. Taruo, *Am. J. Roentgen*, **160**, 1293 (1993).
80. H. H. Schild, W. Weber, E. Boeck, P. Mildenberger, H. Strunk, Duber Ch, P. Grebe, S. Schad mand Fischer and M. Thelen, *Fortschr. Rontgen Str.*, **160**, 218 (1994).
81. W. J. Matchett, D. R. Mc Fanland, D. K. Russell, D. M. Senlor and M. M. Moursc, *Radiology*, **201,** 569 (1996).
82. J. A. Kaufman, S. C. Geller and A. C. Waltman, *Radiology*, **198**, 579 (1996).
83. T. Vehmas and T. Tervahartiala, *Acta. Radiol.*, **37,** 804 (1996).
84. T. Vhemas and T. Markkola, *Acta. Radiol.*, **39**, 223 (1998).
85. F. Fobbe, F. Wacher and S. Wagner, *Eur. Radiol.*, **6**, 224 (1996).
86. T. Stacks, G. Schuhmann-Giampieri, T. Frenzel, H. J. Weinmann, L. Longe and J. Platzek, *Invest. Radiol.*, **29**, 709 (1994).
87. D. J. Spinosa, A. H. Matsumoto, J. F. Angle, K. D. Hagspiel, J. K. Mcgrao and C. R. Ayers, *Radiology*, **209,** 490 (1998).
88. T. Balzer, B. Hamm, T. Staks, H. J. Weinmann, A. Muhler, M. Taupitz and H. P. Niendorf, In: "New Development in Contrast Agent Research", P. A. Rinck and R. N. Muller (Eds.), *European Magnetic Resonance Forum,* Berlin 129 (1995).
89. G. Schumann-Giampieri, M. Mahler, G. Roll, R. Maibencer and S. J. Schmitz, *Chin. Pharmacol.*, **37,** 587 (1997).
90. P. Caravan, J. J. Ellison, T. J. McMurry and R. B. Lauffer, *Chem. Rev.*, **99**, 2293 (1999).
91. V. M. Runge, J. W. Wells and N. M. Williams, *Invest Radiol.*, **30**, 123 (1995).
92. A. Dumont, P. Jacques, P. Qixiu and J. F. Dessreaux, *Tetrahedron Lett.*, **35**, 3707 (1994).
93. W. D. Kim, G. E. Kiefer, F. Maton, K. McMillan, R. N. Muller and A. D. Sherry, *Inorg. Chem.*, **34,** 2223 (1995).
94. G. R. Weisman, E. H. Wong, D. C. Hill, M. E. Rogers, D. P. Reed and J. C. Calabrese, *J. Chem. Soc. Chem., Commun.* 947 (1996).
95. J. Springborg, P. Kofod, C. E. Olsen, H. Toftlund and I. Sotofte, *Acta. Chem. Scand.*, **49**, 547 (1995).
96. X. Y. Wang, T. Z. Jin, V. Comblin, A. Lopez-Mut, E. Meveiny and J. F. Desreanx, *Inorg. Chem.*, **31**, 1095 (1992).
97. H. Gries and H. Miklautz, *Physiol. Chem. Phys. Med.*, NMR, **16**, 105 (1984).
98. J. J. Stezowski and J. L. Hoard, *Isr. J. Chem.*, **24**, 323 (1984).
99. M. B. Inoue, M. Inoue and Q. Fernando, *Acta. Crystallogr.*, Sec C, Cryst. Structure, Commun. **C50**, 1037 (1994).

100. M. B. Inoue, M. Inoue, I. C. Munoz, M. A. Bruck and Q. Fernando, *Inorg. Chim. Acta.*, **209**, 29 (1993).
101. M. B. Inoue, R. E. Navarro, M. Inoue and Q. Fernando, *Inorg. Chem.*, **34**, 6074 (1995).
102. M. B. Inone, M. Inone, P. Oram, Q. Fernando, A. Alexander and E. C. Unger, *Magn. Reson. Imaging*, **12**, 429 (1994).
103. E. Toth, F. Connac, L. Helm, K. Adzamli and A. E. Merbach and J. Ewo, *Inorg. Chem.*, **37**, 2017 (1998).
104. E. Toth, L. Helm, A. E. Merbach, R. Hedinger, K. Hegetschweiler and A. Janossy, *Inorg. Chem.*, **37**, 4104 (1998).
105. E. Toth, Van Leffelen, L. Helm, A. E. Marbach, D. Ladd, K. Briley-Saebo and K. E. Keller, *Magn. Reson. Chem.*, **36**, 5125 (1998).
106. S. Aime, A. Barge, A. Borel, M. Botta, S. Chemerisov, A. E. Merbach, U. Muller and D. Pubanz, *Inorg. Chem.*, **36**, 5104 (1997).
107. R. Ruloff, R. N. Muller, D. Pubanz and A. E. Merbach, *Inorg. Chim. Acta.*, 15, 275-276 (1998).
108. T. Desser, D. Rubin, H. Muller, F. Quing, S. Khodor, S. Zanazzi, S. Young, D. Ladd, J. Wellons, K. Kellor, J. Toner and R. Snow, *J. Magn. Reson. Imaging*, **4**, 467 (1994).
109. A. Bogdanov, R. Weissleder and J. Brady, *J. Adv. Drug Delivery Rev.*, **16**, 335 (1995).
110. B. Raduechel, H. Schmitt-Willich, J. Platzek, W. Ebert, T. Frenzel, B. Misselwitz and H. J. Weinmann, Book of Abstracts, 216$^{th}$ ACS National Meeting, Boston, Aug 23-27, (1998) pMSE-278.
111. Q. Dong, D. R. Hurst, H. J. Weinmann, T. L. Chenevert, F. J. Londy and M. R. Prince, *Invest. Radiol.*, **33**, 699 (1998).
112. J. Tacke, G. Adam, H. Claben, A. Muhler, A. Prescher and R. W. Gunther, *Magn. Reson. Imaging*, **7**, 678 (1997).
113. H. C. Roborts, M. Saeed, T. P. L. Roberts, A. Muhler, D. M. Shames, J. S. Mann, M. Stiskal, F. Demsar and R. C. Brasch, *Magn. Reson. Imaging*, **7**, 331 (1997).
114. H. C. Roberts, M. Saeed, T. P. L. Roberts and R. C. Brasch, *Acad. Radiol.*, **5**, S-31 (1998).
115. C. Curtet, F. Maton, T. Havet, M. Slinkin, A. Mishra, J. F. Chatel and R. N. Muller, *Invest. Radiol.*, **33**, 752 (1998).
116. H. Degani and G. A. Elgavish, *FEBS Lett.*, **90**, 357 (1978).
117. R. K. Gupta and P. J. Gupta, *Magn. Reson.*, **47**, 344 (1982).
118. R. K. Gupta, In: "NMR spectroscopy of Cells and Organs", Vol. 2, R. K. Gupta (ed.), CRC Press 130ca Raton, Florida, USA 1 (1987).
119. M. M. Pike and C. S. Jr. Springer, *J. Magn. Reson.*, **46**, 348 (1982).
120. M. M. Pike, T. W. Smith, E. T. Fossel and C. S. Jr. Springer, *Am. J. Physiol.* **246**, C528 (1984).
121. J. Ren and A. D. Sherry, *J. Magn. Reson.*, Ser B, **111**, 178 (1996).
122. V. Sheshan, M. J. Germann, P. Preisig, C. R. Malloy, A. D. Sherry and N. Bansal, *Magn. Reson. Med.*, **34**, 153 (1995).
123. A. D. Sherry, *J. Alloys and Compound*, **249**, 153 (1997).
124. S. K. Miller and G. A. Elgavish, In: "Biological Magnetic Resonance Spectroscopy", L. J. Berliner and J. Renben (Eds.), Plenum Press, New York, USA 159 (1992).

125. S. C. K. Chu, Y. Xu, J. A. Balschi and C. S. Springer, *Jr. Magn. Reson. Med.*, **13**, 239 (1990).

126. G. A. Elgavish, *Invest. Radiol.*, **24**, 1028 (1989).

127. A. D. Sherry, C. F. G. C. Geraldes and W. P. Cacheris, *Inorg. Chim. Acta.*, **139**, 137 (1987).

128. D. C. Buster, M. M. C. A. Castro, C. F. G. C. Geraldes, C. R. Malloy, A. D. Sherry, T. C. Siemers, *Magn. Reson. Med.*, **15**, (1990) 25.

129. C. R. Malloy, D. C. Buster, M. M. C. A. Castro, C. F. G. C. Geraldes, F. M. H. Jeffrey and A. D. Sherry, *Magn. Reson. Med.*, **15**, 33 (1990).

130. C. F. G. C. Geraldes, A. D. Sherry, G. E. Kiefer, *J. Magn. Reson.*, **97**, 290 (1992).

131. B. Butwell, R. Ramasamy, J. Lazor, A. D. Sherry and C. R. Malloy, *Am. J. Physiol.*, **264**, 41814 (1993).

132. M. M. Pike, C. Su Luo, M. D. Clark, K. A. Kirk, M. Kitakaze, M. C. Madden, E. J. Gragoe and G. M. Pohost, *Am. J. Physiol.*, **265**, 4-2017 (1993).

133. A. F. Xia, J. W. Horton, P. Y. Zhao, N. Bansal, E. E. Babcock, A. D. Sherry and C. R. Malloy, *J. Appl. Physiol.*, **76**, 1507 (1994).

134. J. C. G. Bunzli and C. Piquet, *Chem. Rev.*, **102**, 1897 (2002).

135. D. Parker, *Coord. Chem. Rev.*, **205**, 109 (2000).

136. D. Parker, Dickins, H. Puschmann, C. Crossland and J. A. K. Howard, *Chem. Rev.*, **102**, 1997 (2002).

137. S. Aime, M. Botta, J. L. Bruce, V. Mainevo, D. Parker and E. Terreno, *Chem. Commun.*, 115 (2001).

138. M. Tsukube, S. Shinoda, J. Uenishi, M. Shiode and O. Yonemitao, *Chem. Lett.*, 969 (1996).

139. M. Tsukube, S. Shinoda, *Tetrahedron Lett.*, **42**, 7583 (2001).

140. Y. Kojima, M. Takemura, K. Yamato, M. Doe, M. Watanabe, H. Miyake, T. Kikunaga and N. Yanagihara, *Bull. Chem. Soc.*, Japan, **74**, 707 (2001).

141. Y. Kojima, M. Watanabe, T. Hasegawa and H. Miyake, *Chem. Lett.*, 4 (2001).

142. K. Kabuto, R. Hazama, K. Umakoshi, C. Kabuto and Y. Sasaki, *Chem. Commun.* 15 (1996).

143. I. Hemmila, T. Stahlberg and P. Mottram, "Bioanalytical Applications of Labeling Technologies", Wallac Dy, Turku (1995).

144. G. Mathis, In: "Rare Earths", R. S. Puche and P. Caro (Eds.), Editorial complutense, S A, Madrid 285 (1998).

145. H. Takalo, V. M. Mukkala, L. Merio, J. C. Roderiguez-ubis, R. Sedano, O. Junnes and E. Brunet, *Helv. Chim. Acta.*, **80**, 372 (1997).

146. J I. Bruce, R. S. Dickins, L. J. Govenlock, S. Lopinski, T. Gunnlangsson, M. P. Lowe, D. Parker, R. D. Peacock, J. J. Perry, S. Aime and M. Botta, *J. Am. Chem. Soc.*, **122**, 9674 (2000).

147. S. I. Klink, P. Oude Alink, L. Grove, F. G. A. Peters, J. W. Hofstraat-Geurtsf and F. C. G. M. Van Veggal, *J. Chem. Soc.*, Perkin Trans, **2**, 363 (2001).

148. S. N. Misra and S. O. Sommerer, *Appl. Spectrosc. Rev.*, **26**, 152 (1991); *Rev. Inorg. Chem.*, **12**, 157 (1992); *Can. J. Chem.*, **72**, 42 (1992).

149. S. N. Misra and K. John, *Appl. Spectrosc. Rev.*, **28**, 285 (1993).

150. R. S. Dickins, J. A. K. Howard, C. L. Manpin, J. M. Maloney, D. Parker, R. D. Peacock, J. P. Riehl and G. Siligardi, *New J. Chem.*, 891 (1998).

151. Y. Shen, T. Riedener and K. L. Bray, *Phys. Rev.*, **B61**, 11460 (2000).
152. M. A. Shubhan, T. Suzuki and S. Kaizaki, *J. Chem. Soc.*, Dalton Trans, 492 (2001).
153. J. C. G. Bunzli, M. Elhobiri, R. Scopelliti and C. Piguet, *J. Am. Chem. Soc.*, **121**, 10747 (1999).
154. J. C. G. Bunzli and C. Piquet, In: " Encyclopedia of Materials", *Science and Technology*, K. H. N. Bushow, R. W. Cahn, M. C. Flemming, B. Ilschner, E. J. Kramer and S. Mahajan (Eds.), Pergamon, Elsevier Science Ltd., Oxford, (2001).
155. Y. J. Ji, Z. H. Wang, J. L. Li and S. H. Peng, *J. Health Toxicol.*, **8**, 165 (1994).
156. Y. X. Nie, Y. L. Chen, P. L. Zhao, X. H. Wang, C. J. Zhang and Z. X. Li, *J. Chinese Rare Earth Soc.*, **8**, 350 (1990).
157. X. M. Li, D. Q. Zhu, P..L. Zhao and J. Z. Ni, *Sci. Bull.* (Chinese), **40**, 2044 (1995).

